# Iterative orthology prediction uncovers new mitochondrial proteins and identifies C12orf62 as the human ortholog of COX14, a protein involved in the assembly of cytochrome *c *oxidase

**DOI:** 10.1186/gb-2012-13-2-r12

**Published:** 2012-02-22

**Authors:** Radek Szklarczyk, Bas FJ Wanschers, Thomas D Cuypers, John J Esseling, Moniek Riemersma, Mariël AM van den Brand, Jolein Gloerich, Edwin Lasonder, Lambert P van den Heuvel, Leo G Nijtmans, Martijn A Huynen

**Affiliations:** 1 Centre for Molecular and Biomolecular Informatics, Radboud University Nijmegen Medical Centre, Nijmegen, 6500 HB, The Netherlands; 2 Nijmegen Centre for Mitochondrial Disorders at the Department of Pediatrics, Radboud University Nijmegen Medical Centre, Nijmegen, 6500 HB, The Netherlands; 3 Theoretical Biology and Bioinformatics, Utrecht University, Utrecht, 3584 CH, The Netherlands; 4 Department of Biochemistry, Nijmegen Centre for Molecular Life Sciences, Radboud University Nijmegen Medical Centre, Nijmegen, 6500 HB, The Netherlands; 5 Nijmegen Proteomics Facility, Department of Laboratory Medicine, Laboratory of Genetic, Endocrine and Metabolic Diseases, Radboud University Nijmegen Medical Centre, Nijmegen, 6500 HB, The Netherlands

## Abstract

**Background:**

Orthology is a central tenet of comparative genomics and ortholog identification is instrumental to protein function prediction. Major advances have been made to determine orthology relations among a set of homologous proteins. However, they depend on the comparison of individual sequences and do not take into account divergent orthologs.

**Results:**

We have developed an iterative orthology prediction method, Ortho-Profile, that uses reciprocal best hits at the level of sequence profiles to infer orthology. It increases ortholog detection by 20% compared to sequence-to-sequence comparisons. Ortho-Profile predicts 598 human orthologs of mitochondrial proteins from *Saccharomyces cerevisiae *and *Schizosaccharomyces pombe *with 94% accuracy. Of these, 181 were not known to localize to mitochondria in mammals. Among the predictions of the Ortho-Profile method are 11 human cytochrome *c *oxidase (COX) assembly proteins that are implicated in mitochondrial function and disease. Their co-expression patterns, experimentally verified subcellular localization, and co-purification with human COX-associated proteins support these predictions. For the human gene *C12orf62*, the ortholog of *S. cerevisiae COX14*, we specifically confirm its role in negative regulation of the translation of cytochrome *c *oxidase.

**Conclusions:**

Divergent homologs can often only be detected by comparing sequence profiles and profile-based hidden Markov models. The Ortho-Profile method takes advantage of these techniques in the quest for orthologs.

## Background

From the publication of the first genome sequences, the identification of orthologs has been a central theme in comparative genomics [1]. Functional genomics as well as genome annotation have greatly benefited from the wealth of experimental data available for model species. To formulate hypotheses about gene functions in remaining organisms, including human, it is necessary to unambiguously resolve the phylogenetic relationships among homologs [2]. The detection of homology, and therewith also orthology, can be crippled by the lack of detectable sequence similarity. Large evolutionary distances, high rates of sequence evolution, low complexity regions and short protein length can preclude homology detection by pairwise sequence similarity approaches such as FASTA or BLAST [3,4]. More sensitive methods can detect remote homologs by replacing general amino acid similarity matrices with position-specific vectors of amino acid frequencies in a profile-to-sequence comparison (PSI-BLAST) [5] or in a profile-to-profile comparison [6]. Profile-based hidden Markov models (HMM) additionally contain information about insertions and deletions and enable the detection of even more remote homologs [7], especially in HMM-to-HMM comparisons [8].

Homology is widely used to transfer information on protein function from model species. For example, homologs of yeast mitochondrial proteins have been used to predict mitochondrial proteins in human [9], and homology-based presence-absence patterns of genes have been applied to subcellular localization prediction [10]. However, assigning subcellular localization based on solely the homology criterion leads to a high false discovery rate of 38% [11]. For larger evolutionary distances (homology with proteins from *Rickettsia prowazekii*, a bacterial relative of mitochondria) inferring subcellular localization based on the homology criterion yields an estimated 73% false positives [11], rendering homology of limited value for localization prediction. Additionally, evolutionary events such as gene duplications often prompt a change of subcellular localization, while one-to-one orthologs tend to localize to the same compartment [12]. This suggests that orthology relationships are more reliable to infer the localization of proteins than just homology relationships. Indeed, manual analyses of orthology relationships between mitochondrial protein complexes from yeast and human [13-17] and automated analyses of complex membership in general [18] have confirmed that orthologous proteins remain involved in the same protein complexes. Importantly, profile-based methods have detected homology between proteins from the same mitochondrial complex in various species that went undetected by pairwise sequence comparison methods. For example, profile-based methods were crucial in the detection of a number of subunits of the NADH:ubiquinone oxidoreductase (complex I) [13,14,17,19,20], the mitochondrial ribosome [16,21] and the mitochondrial Holliday junction resolvase domain [22]. Such *ad hoc *procedures have, however, not been systematically assessed for their quantitative contribution and qualitative reliability in the large-scale detection of orthology relationships.

To include profiles in large-scale orthology inference, we introduce a three-phase procedure (Ortho-Profile) that applies reciprocal best hits at the sequence-to-sequence, the profile-to-sequence and finally the profile-based HMM-to-HMM level. To test the quality of our orthology assignment, we use protein subcellular localization, an important aspect of protein function that has been established experimentally in a number of species and is amenable to large-scale analysis. Mitochondrial localization has been established on a genome-wide scale (as well as in small-scale experiments) for proteins in *Saccharomyces cerevisiae *[23] and *Schizosaccharomyces pombe *[24]. The mitochondrial proteins of these distant eukaryotic relatives have previously been used as models for mammalian mitochondrial proteins and for systematic predictions of human mitochondrial disease genes [25].

In the analysis presented here the fungal mitochondrial proteins serve as a starting point for large-scale orthology prediction in human. Of the one-to-one orthologs predicted between fungal mitochondrial proteins and human, 181 proteins have to date not been shown to localize to mitochondria in human (Table S6 in Additional file 1). For 15 proteins we find corroborating evidence for their mitochondrial localization using a probabilistic analysis of genome-wide data from Pagliarini and co-workers [11].

Cytochrome *c *oxidase (COX) is a 13-subunit enzyme complex in mammals that catalyzes the terminal step of the mitochondrial respiratory chain, accepting electrons from cytochrome *c *and passing them to molecular oxygen, producing water. Early biochemical analyses of COX defects in human have suggested that most COX deficiencies stem from decreased stability or failure to complete assembly of the holoenzyme [26,27]. Defects in the assembly process cause severe neuromuscular disorders in human, the so-called mitochondrial encephalomyopathies [28]. The identification of human orthologs of yeast COX assembly factors has helped to identify pathogenic mutations in human [29], including the first mutations in a nuclear gene involved in human COX deficiency, *SURF1 *[30,31]. The Ortho-Profile method contributed to the recent identification of a mutation in the *COA5 *(previously *C2orf64*) gene that leads to COX deficiency [32], but causal genes for many other disease cases are still not known. In this work we predict 11 candidates for COX assembly factors and confirm the mitochondrial localization of four human candidate proteins. The high co-expression of the majority of the candidates with mammalian oxidative respiratory complexes as well as co-purifications of the candidates with COX-associated proteins give additional weight to our predictions. We experimentally confirm the role of C12orf62 as a COX assembly factor binding to COX1 and acting as its translation regulator.

## Results and discussion

**Figure 1:**
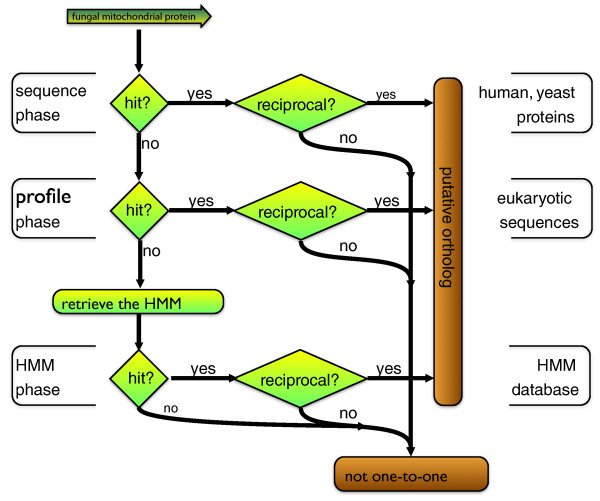
**Ortho-Profile, the three-phase method for identifying distant orthologs**. Orthologous human and fungal proteins were determined by means of bi-directional best hits at the sequence-to-sequence, profile-to-sequence and HMM-to-HMM levels. The pipeline detects distant orthologs owing to increasingly sensitive methods applied in subsequent phases (see Materials and methods for details).

We carried out large-scale prediction of human orthologs of mitochondrial proteins from the fungal model species *S. cerevisiae *and *S. pombe*, employing the reciprocal best hit approach to sequences, sequence profiles and profile-based HMMs. We designed the iterative Ortho-Profile method that includes subsequent phases of increasing sensitivity: sequence-to-sequence (BLAST) [3], profile-to-sequence (PSI-BLAST) [5] and HMM-to-HMM search algorithms (HHSearch) [8] (Figure 1 and Materials and methods). The three phases ensure high accuracy in inference, both of orthologs similar in their sequence (in the sequence-to-sequence phase) and of less similar, faster evolving sequences (HMM-to-HMM phase). Ortho-Profile thus identifies more divergent orthologs in subsequent phases, while maintaining specificity for members of large gene families in the first phase.

In the sequence-to-sequence phase [5] the method uses BLAST to search for a homolog of a *S. cerevisiae *protein in human and tests whether the human protein is also the reciprocally best (most similar and statistically significant) homolog in *S. cerevisiae*. If no homolog is found in the sequence-to-sequence phase (see Materials and methods for details), a profile search is initiated to increase the sensitivity. In the profile-to-sequence search PSI-BLAST [5] is employed, using the *nr *database of protein sequences (Materials and methods). If no statistically significant (E < 0.01) human homolog of a *S. cerevisiae *mitochondrial protein has been found among the PSI-BLAST hits in the first iteration, subsequent iterations extend the profile with (non-human) homologs that have been found (inclusion threshold 0.005). Up to three iterations are carried out to find a statistically significant human homolog. If a human homolog of the *S. cerevisiae *mitochondrial protein has been identified, a reciprocal search is carried out. The reciprocal search starts with the human protein to find a yeast homolog, again in up to three PSI-BLAST iterations. To satisfy the bi-directional best hit criterion, the first statistically significant *S. cerevisiae *gene to be encountered in the reciprocal search phase should be the original query gene. Finally, if no bi-directional best hit has been detected in the profile-to-sequence phase, a profile-to-profile search is carried out to increase sensitivity even further. The HMM phase operates on the databases of HMMs built for each protein sequence from fungal and human genomes using homologs in a wide range of species (see Materials and methods for details). The profile-representing HMM for the *S. cerevisiae *protein is retrieved from an HMM database. Subsequently, the database of HMMs that represent human proteins is searched for a homologous HMM using HHsearch [8]. Analogous to the first phases, if a homologous best hit is found, a reciprocal HMM search is carried out, by comparing the HMM that contains the human protein with the *S. cerevisiae *HMM database. The same iterative procedure is carried out for *S. pombe *mitochondrial proteins.

To test the procedure, we collected experimentally determined mitochondrial proteomes from two model fungal species, *S. cerevisiae *(1,056 proteins), and *S. pombe *(718 proteins). This resulted in the identification of 598 human genes as putative orthologs of fungal mitochondrial proteins (reciprocal best hits). In any of the three phases, *S. cerevisiae *accounts for 429 proteins of the reciprocal best hits with human and *S. pombe *for 497 proteins, while 328 orthologs are best reciprocal hits in both species. For an additional 246 fungal proteins, homologs were found in the human genome, but the human homologs were not reciprocal best hits in the fungi. For the remaining fungal genes no homologs were found. The two most sensitive phases of the method, profile-to-sequence and the HMM-to-HMM, provide 22% of all identified orthologous pairs (Table 1).

**Table 1:** Subcellular localization of human orthologs of yeast mitochondrial proteins

	Sequence	Profile	HMM	Total (localization)
Mitochondrial	338	37	42	417 (192^a^)
Non-mitochondrial	63	10	19	92 (20^a^)
Unknown	59	8	22	89
Total (method)	460	55	83	598

Subcellular localization of human orthologs of yeast mitochondrial proteins. In columns, we list numbers of proteins that contributed by sequence-to-sequence, sequence-to-profile and HMM-to-HMM method phases. Rows present known subcellular localization according to annotation based on the experimental evidence in human or mouse (Table S6 in Additional file 1). ^a^Corroborated by data from both human and mouse independently.

### Accuracy of the pipeline

A number of benchmarks indicate the high quality of the orthology prediction. Firstly, the method recovers all but one manually annotated human ortholog of the small and the large subunits of the fungal mitochondrial ribosome, 51 proteins in total. Also, for all but one *S. pombe *mitoribosomal fusion protein, orthology relationships were resolved correctly when compared to the phylogeny-based orthology prediction [16]. Benchmarking with a manually curated ortholog inventory of *S. pombe *and *S. cerevisiae *[33] shows that orthologs of human proteins in the two yeasts are consistent with the curated inventory in 95% cases, that is, the manually curated *S. pombe-S. cerevisiae *orthologs are orthologous to the same human protein (see Materials and methods for details). A domain composition analysis using PFAM [34] reveals that 84% of the predicted orthologs have an identical domain composition in human and fungi (504/598, including 5% of orthologs that have no detectable protein domains), corroborating the orthology prediction. However, domain composition data on their own, without inferred orthology, are not a strong predictor of subcellular localization (Materials and methods).

There are 417 human orthologs of fungal mitochondrial proteins that localize to mitochondria according to annotations based on experimental data in human and mouse or are part of a compendium of mammalian mitochondrial proteins that is based on integrated experimental data and sequence-based predictions [11] (Table 1; Table S6 in Additional file 1). This encompasses 70% of the complete set of 598 orthologs that we identified, with 192 proteins (32%) corroborated by both human and mouse localization data. Among the 181 proteins (30%) that are not annotated as mitochondrial in mammals, 92 proteins (15% of all orthologs) are annotated with another subcellular compartment (Table 1). The non-mitochondrial localization may, at least for some proteins, be an indication of dual localization, a phenomenon not uncommon in eukaryotes [35]. Only 20 proteins (3%) have been found in the same non-mitochondrial compartment in both human and mouse (Table 1). The limited number of non-mitochondrial proteins among mammalian orthologs of fungal mitochondrial proteins demonstrates the predictive power of orthology prediction with respect to subcellular localization.

We tested if the reciprocal best hit as well as the homology criteria are actually both necessary for a correct prediction of subcellular localization. For human homologs of fungal mitochondrial proteins that are not reciprocal best hits (212 proteins), and therewith not one-to-one orthologs, only 38 are mitochondrial (18% of non-orthologs compared to 70% orthologs). Out of 212 non-orthologous proteins, 75 are known to localize to other subcellular compartments (35% of the non-orthologous homologs compared to 3% of the orthologs). Among orthologs of fungal mitochondrial proteins there are 4.5 more mitochondrial than non-mitochondrial human proteins (Table 1). For homologs there are two times less mitochondrial than non-mitochondrial ones (Table S7 in Additional file 2), implying that a homology relationship on its own does not predict localization as accurately as orthology. High conservation of localization also holds for more divergent orthologs (detectable only with profile and HMM methods), where homology has limited predictive power (Figure S4 in Additional file 2).

We also evaluated the reciprocal best hit criterion, without homology or a statistically significant similarity required. Protein pairs that fall outside the significance threshold in the sequence-to-sequence comparison (E ≥ 0.01) might still be reciprocal best hits based on their raw BLOSUM similarity scores. Among these reciprocal best hits only 23% of human proteins were annotated as mitochondrial in human. Thus, both homology and reciprocal best criteria are important for high-quality localization prediction.

### Orthologs of yeast proteins involved in cytochrome *c *oxidase assembly

Improved, profile-based orthology detection can be used to predict new organellar proteins in human (see above), but it is also invaluable for predicting protein function. Examination of the predicted orthology relations between the proteins of fungal mitochondria and those of human (Table S6 in Additional file 1) revealed a number of cases in which the fungal protein was known to be involved in the assembly of COX, while there was no (predicted) function for the human protein. COX assembly factors and maintenance proteins are rapidly evolving, mostly short proteins (< 100 amino acids) whose evolutionary history and orthologs in other species have often eluded detection due to limited sequence similarities. From databases and literature we collected 42 COX assembly factors in *S. cerevisiae *(splicing factors, transcription and translation activators and regulators, proteins involved in COX membrane insertion, assembly and maintenance; Materials and methods; Table S5 in Additional file 2). From this list, 11 predicted orthologs in human had not previously been implicated in COX assembly in mammals (Table 2). Data on co-expression with subunits of respiratory chain complexes in mammals [36] nevertheless support the involvement of these COX assembly candidates in oxidative phosphorylation in human (Table 2). The co-expression of the putative COX-assembly proteins with subunits of oxidative phosphorylation is high (with average integrated probability of co-expression at 0.67) compared to co-expression of the remaining mitochondrial proteins (average 0.34, *n *= 1180, different at P < 0.01, two-tailed Wilcoxon rank sum test) and non-mitochondrial proteins (average 0.10, *n *= 15,036, *P *< 0.0001). As a negative control for our method we examined the genome of the anaerobic stramenopile *Blastocystis hominis*, a species with a mitochondrion and a mitochondrial genome, but without a COX complex. No orthologs of the 11 postulated COX assembly factors could be detected in that species (see Supplemental Methods in Additional file 2 for details).

**Table 2:** Candidate COX assembly factors

Yeast			Human			
		
Gene	Description	Phase	Gene	Targeting signal	Mitochondrial localization	OXPHOS co-expression
*COX14 *	Negative translation regulation of COX1 translation	HHM	*C12orf62*	No	+	0.93
*COX20*	Proteolytic processing of Cox2p and its assembly into COX	Profile	*FAM36A*	No	+^a^	0.63
*COX23*	Cytochrome oxidase assembly	Sequence	*CHCHD7*	No	*ND*	0.63
*COX24*	Required for accumulation of spliced *COX1 *mRNA	HMM	*AURKAIP1*	Yes	+	0.91
*COA1*	Cytochrome oxidase assembly	HMM	*C7orf44*	Yes	+	0.73
*COA3*	Negative regulation of COX1 subunit	HMM	*CCDC56*	No	+^b^	0.92
*MSS51*	UTR translation COX1 regulation	Profile	*ZMYND17*	Yes	*ND*	0.01
*PET100*	Assembly of COX	Profile	*PET100/LOC100131801*	No	+^b^	*ND*^c^
*PET117*	Assembly of COX	Sequence	*PET117/LOC100303755*	No	+	*ND*
*PET191*	Assembly of COX	Sequence	*COA5/C2orf64*	No	*ND*^d^	0.55
*PET309*	Translation activator of COX1	Profile	*PTCD1*	Yes	*ND*	0.48
*YMR244C-A*	Putative protein of unknown function	Sequence	*C1orf31*	Yes	+^2^	0.8

**Figure 2:**
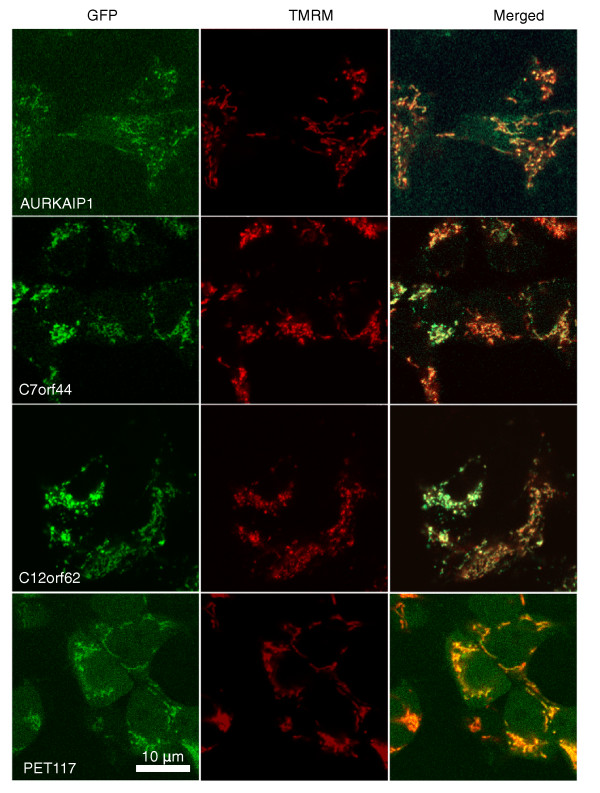
**Localization of the predicted COX assembly factors in human mitochondria**. The figure shows co-localization of AURKAIP1, C7orf44, C12orf62 and PET117 proteins with the mitochondrial marker tetramethyl rhodamine methyl ester (TMRM). We performed live cell imaging of HEK293 cells expressing GFP-tagged genes (left panels) loaded with the mitochondrial marker TMRM (middle panels). Superimposed images are shown on the right. Yellow indicates the GFP-TMRM overlap.

Human orthologs of yeast COX assembly factors inferred with Ortho-Profile that have not been previously linked to COX assembly in mammals. The targeting signal is predicted with TargetP [66]. Confirmed mitochondrial localization is marked with a plus sign (+; see also Figure 2). Integrated probability of co-expression with oxidative phosphorylation (OXPHOS) complexes in mammalian cells from [36]; ND, no data. ^a^GFP-validated mitochondrial localization; ^b^protein presence in pure mitochondrial fractionations from [11]. ^c^Co-expresses with OXPHOS subunits in *Drosophila melanogaster *(data from STRING 9.0 [69]; Supplemental Methods in Additional file 2). ^d^Our study showed that the mutation causes COX assembly defect and mitochondrial cardiomyopathy [32].

### Four predicted COX assembly factors are targeted to, and reside in, mitochondria

We successfully obtained human embryonic kidney 293 (HEK293) cells that stably express the green fluorescent protein (GFP)-tagged variants of five of the human COX assembly candidates listed in Table 2: *PET100 *(*LOC100131801*), *PET117 *(*LOC100303755*), *AURKAIP1, C7orf44*, and *C12orf62 *(Materials and methods). The transfected cells were loaded with tetramethyl rhodamine methyl ester (TMRM), a fluorescent dye that localizes to mitochondria. Four of the proteins (AURKAIP1-, C7orf44-, C12orf62- and PET117-GFP) co-localize with the mitochondrial marker (Figure 2). While the confocal microscope image analysis of PET100 did not allow assigning the protein to a specific compartment, a cellular fractionation experiment shows that PET100 is present in the intracellular membrane (Figure S2 in Additional file 2). Additionally, our re-analysis of raw liquid chromatography tandem mass spectrometry (LC-MS/MS) data [11] of isolated mouse heart mitochondria, previously hindered by the absence of PET100 from protein catalogs, has identified PET100 in pure mitochondrial extracts (Supplemental Methods in Additional file 2).

### COX-associated proteins co-purify with predicted COX assembly factors

Tandem affinity purifications (TAPs) were carried out to identify proteins that co-purify with COX assembly candidates. We generated HEK293 T-REx cells that inducibly express the predicted COX assembly factors with a carboxy-terminal TAP tag. After a 24 h induction, cell lysates were affinity purified and eluates were analyzed using nanoLC-MS/MS to identify purified proteins. When using PET100, PET117, C7orf44 and C12orf62 as bait, the COX17 protein was co-purified. COX17 was not co-purified without induced expression of these proteins, or with a control set of non-COX assembly mitochondrial proteins (Materials and methods). COX17 is an assembly factor known to play a role in copper transfer [37] and is a part of a larger 150 kDa complex [38]. The subunit VIIa of COX (encoded by the *COX7A2 *gene) was specifically co-purified with PET100, corroborating the conserved interaction of fungal Pet100p-subunit VIIa that takes place in the inner mitochondrial membrane of yeast [39] (Supplemental Methods in Additional file 2). In addition, the LC-MS/MS analysis of C7orf44-TAP purifications identifies C1orf31, a putative assembly factor and a paralog of the COX6B subunit. Co-purified COX-associated proteins are shown in Table 3. While more proteins co-purify with the assembly candidates (see Materials and methods and Table S4 in Additional file 2 for the list of all co-purified mitochondrial proteins) these COX proteins were not co-purified for control proteins.

**Table 3:** Proteins co-purified with candidate COX assembly factors

	Purified		
	
Tagged	COX7A2	COX17	C1orf31
C7orf44-TAP	-	+	+
PET100-TAP	+	+	-
PET117-TAP	-	+	-
C12orf62-TAP	-	+	-
AURKAIP1-TAP	-	-	-

COX-associated proteins co-purified with the predicted COX assembly factors but not with control proteins. COX7A2 is COX subunit VIIa, COX17 is a known COX assembly factor and C1orf31 is a predicted assembly factor. In all induced samples the bait protein was identified (Materials and methods).

### C12orf62 overexpression reduces COX protein levels

**Figure 3:**
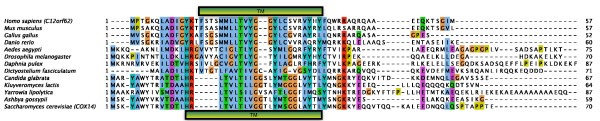
**Multiple sequence alignment of C12orf62 with its orthologs, including the fungal COX14**. The transmembrane regions, predicted by TMHMM [66], are marked for the human (top) and yeast (bottom) sequences. The alignment was made using CLUSTAL-W [67] and visualized with Jalview [68].

**Figure 4:**
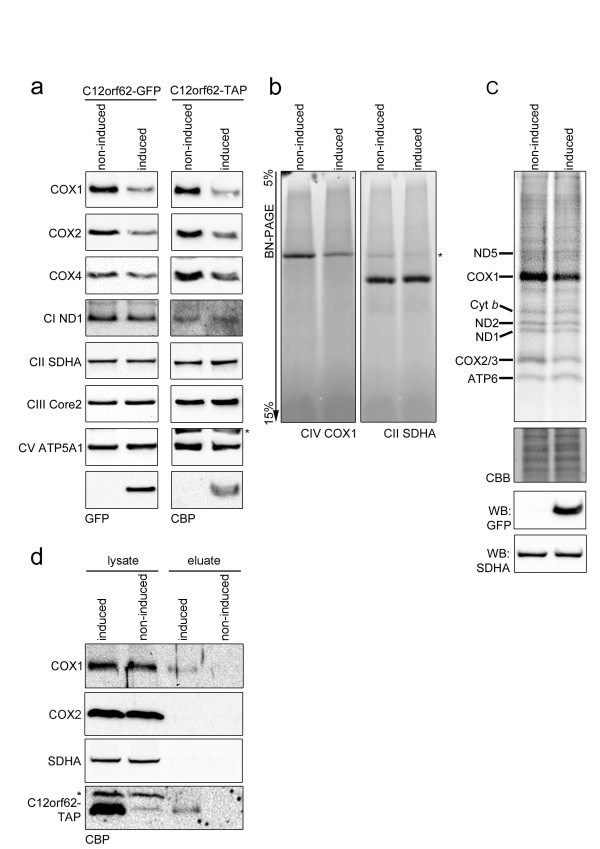
**C12orf62 is a novel COX assembly factor**. C12orf62 binds to COX1 and overexpression of C12orf62-GFP and C12orf62-TAP results in reduced COX levels and activity. **(a) **Protein levels of subunits of five respiratory chain complexes. HEK293 cells were induced by doxycycline to overexpress C12orf62-GFP and -TAP (Materials and methods and Additional file 2). SDS-PAGE blots were immunodecorated with indicated antibodies. **(b) **The effect of *C12orf62 *overexpression on the COX holocomplex. Blue Native (BN)-PAGE analysis followed by immunodetection of complex IV (CIV) subunit COX1 and complex II subunit SDHA from *C12orf62-GFP *overexpressing HEK293 cells. **(c) **Overexpression of C12orf62-GFP affects levels of newly synthesized COX proteins. ^35^S labeling of mitochondrial translation products from HEK293 cells overexpressing C12orf62 versus non-induced control cells. To confirm equal loading, gels were rehydrated and stained with Coomassie Brilliant Blue G-250 (CBB). Expression of the transgene and protein loading was confirmed with SDS-PAGE followed by western blotting (WB) and incubations with indicated antibodies. The loading was carried out twice (Table S9 in Additional file 2). **(d) **C12orf62 interacts with COX1. C12orf62-TAP was affinity purified from HEK293 cells. The purified C12orf62-TAP (eluates) were analyzed with SDS-PAGE and western blotting for co-purified proteins by probing the membranes with the indicated antibodies. Non-induced cells were used as a control. The efficiency of the pull-down was tested with the TAP-tag recognizing CBP (calmodulin binding peptide) antibody. Asterisks denote signals from previous incubations.

While the specific molecular function of many COX assembly factors is unknown, *COX14 *has been identified as a negative regulator of COX in *S. cerevisiae*, down-regulating *COX1 *expression [40]. We tested the effect of the overexpression of *C12orf62*, the predicted human ortholog of *COX14 *(see Figure 3 for the alignment), on the COX levels in HEK293 cells. The doxycycline-induced overexpression of both C12orf62-GFP and -TAP proteins yields lower protein levels of COX1, COX2 and COX4 without severely affecting the mitochondria-encoded ND1 (complex I subunit) and other oxidative phosphorylation (OXPHOS) subunits (Figure 4a). The reduced protein levels of other subunits that join COX1 later in the assembly process (mitochondria-encoded COX2 as well as nuclear-encoded COX4) [41] may be an effect of rapid protein degradation, as has been observed for COX2 in compromised COX1 synthesis [42]. Aside from the lower levels of the individual COX proteins, C12orf62 overexpression also results in lower levels of the COX holocomplex as revealed by Blue Native (BN)-PAGE analysis (Figure 4b). We additionally performed *in vivo *labeling studies to test whether C12orf62 overexpression influences the translation of the COX1 protein. ^35^S labeling of mitochondrial translation products reveals lower levels of newly synthesized COX1 (as well as COX2/3) in induced cells, but does not interfere with the mitochondrial translation in general (Figure 4c).

### C12orf62 is complexed to COX1

The C12orf62-TAP affinity purification was carried out and analyzed with SDS-PAGE and western blotting. Based on the observation that Cox1 in yeast is found in a complex with COX14 [40,43], we also tested for a possible co-purification of the human COX1 protein with C12orf62, detecting COX1 in the C12orf62-TAP eluate. Despite low C12orf62-TAP protein levels (possibly caused by limited accessibility of the TAP-tag) the eluate revealed specific interaction with COX1 (Figure 4d).

## Conclusions

We introduce the Ortho-Profile method that identifies orthologs in the sequence homology 'twilight zone', where short proteins, high rates of sequence evolution and composition biases make genes' evolutionary relationships difficult to infer. Ortho-Profile, owing to the iterative approach combined with the high sensitivity of profile-to-sequence and HMM-to-HMM searches, allows detection of even remote orthologs and thus complements other large-scale orthology prediction systems. In-paranoid [44], Ortho-MCL [45] and phylogeny-based orthology determination [46,47] are applicable for orthology reconstruction when homology is detectable at the protein sequence level. For more divergent proteins, homology detection based on sequence-profiles is sometimes used (including the presence of PFAM domains) or overlooked. We show that homology does not predict subcellular localization as accurately as orthology, and that orthology confidently predicts localization, even when it is inferred even for very divergent sequences. With no detectable homology in the sequence-to-sequence comparisons, reciprocal best hits at the profile-to-sequence and HMM-to-HMM levels enable orthology inference. Conserved subcellular localization indicates that the quality of inferred orthologs does not reduce significantly for very divergent genes in the 'orthology twilight zone' (Table 1 and Figure S4 in Additional file 2), confirming the accuracy of the presented approach.

We employ subcellular localization, an essential aspect of protein function, to evaluate the quality of orthology prediction. Localization has been established experimentally on the complete proteome-scale in an unbiased manner, independently in both human and fungi, and serves as a proxy for conservation of protein function that is amenable for large-scale analysis. We show that the identification of orthologs is instructive for establishing protein localization and the role of proteins in the cell. The Ortho-Profile method derives 181 new orthology relations between fungal mitochondrial proteins and human (including 59 from profile and HMM phases; Table 1) that have not been previously linked with mitochondria in mammals. As knowledge about the human mitochondrial proteome is not yet complete, many of these orthologs may localize to the organelle, their detection obscured by limited tissue distribution, low protein expression or absence from gene catalogs. These candidates were re-analyzed using a Bayesian framework, integrating the orthology data with co-expression, targeting signal prediction and proteomics data [9,11]. The analysis suggests 15 additional candidate proteins for the inclusion in the human mitochondrial proteome (Materials and methods; Table S3 in Additional file 2). This is an underestimate of the real number of novel mitochondrial proteins, as even proteins for which we confirm the mitochondrial localization by GFP tagging (C7orf44 and PET117) do not receive strong support from other types of genome-wide data and do not reach the threshold that corresponds to a 10% false discovery rate.

We predict the human COX assembly candidates based on orthology with *S. cerevisiae *proteins and provide experimental validation for their subcellular localization (Table 3). An important difference between mitochondrial COX genes of mammals and of yeast is that the latter include introns, and a number of COX assembly factors in yeast are actually involved in splicing. Consistently, we do not detect orthologs of these splicing genes in the human genome (Supplemental Methods in Additional file 2). In contrast there appears to be more conservation at the level of translation. *SURF1*, the human ortholog of yeast *SHY1*, is a known COX assembly factor [30,31] that participates in *COX1 *translation. The Ortho-Profile method identifies orthologs of multiple genes that control the *COX1 *translation process in fungi (*COA1-C7orf44, COA3-CCDC56, COX14-C12orf62*; Table 2). The proposed role of the human orthologs in *COX1 *translation is corroborated by their mitochondrial localization (Table 3) and the observed negative effect of *C12orf62 *overexpression on *COX1 *translation, as well as the physical association of the latter two proteins (Figure 4). Additional genes have been implicated in *COX1 *translation in human: *TACO1 *[42] and *PET309*'s homolog pentatricopeptide repeat-containing *LRPPRC *[48-50]. Our method identifies pentatricopeptide repeat-rich protein *PTCD1 *as an ortholog of the fungal *PET309 *gene. *PTCD1 *has been recently implicated in negative regulation of leucine tRNA levels, as well as negative regulation of mitochondria-encoded proteins and COX activity [51].

Recently, the identification of human orthologs of yeast COX assembly factors allowed us to prioritize *C2orf64*/*COA5 *(Table 2) as a candidate gene for a neonatal cardiomyopathy [32]. Additionally, while this work was under review, a report on neonatal lactic acidosis was published [52] that supports our prediction and experimental confirmation of C12orf62 as a COX assembly factor. Of note, the authors argue that C12orf62 is a vertebrate-specific protein, while we show that it is orthologous to COX14. These discoveries signify the relevance of orthology prediction using profile-based approaches, such as Ortho-Profile, for biomedical research.

## Materials and methods

### Orthology pipeline

The pipeline uses multiple homology detection methods to establish reciprocal best hits between a set of query genes (representing yeast mitochondrial proteomes) and human genes. The less divergent members of large protein families with multiple members per genome may hinder correct identification of orthologs if only the profile-based phases (profile-to-sequence or HMM-to-HMM) are used; thus, the pipeline was designed with three phases of increasing sensitivity, proceeding to a subsequent phase only if no orthology was detected in the previous phase.

In the first stage a BLAST search is performed on the human gene complement, using a yeast mitochondrial protein as the query sequence. If a significant similarity (E < 0.01) has been found, a reverse search is performed with the best hit (lowest E-value), to establish whether the human homolog is a statistically significant reciprocal best hit of the yeast mitochondrial protein (E < 0.01). In the case that no homolog is found, the search proceeds to the profile-to-sequence (PSI-BLAST) stage. In this second stage, three iterations of PSI-BLAST are run (E < 0.01, profile inclusion threshold 0.005), using the complete *nr *database for the construction of profiles. The first statistically significant homolog (E < 0.01) from the earliest PSI-BLAST iterations is selected, even if in the following iterations homologs with lower E-values are detected. In the last Ortho-Profile phase, a profile-based HMM that represents the query sequence is retrieved. Subsequently, the HMM is compared to an HMM database that represents the complete genome of the subject species. The best hit (based on the E-value) is used to establish reciprocity, analogously to the previous stages (E < 0.01).

### HMM profile construction

The database of profiles for human and *S. cerevisiae *that were constructed using the HHPred toolkit, version 1.5.1.1 [8] were downloaded from [53]. For the profile construction of *S. pombe*, default options were used for the iterative multiple sequence alignment building stage PSI-BLAST (2.2.18) [5], running for eight cycles or until convergence. After each cycle of the standard PSI-BLAST algorithm, portions with insufficient similarity to the sequences in the multiple sequence alignment were pruned, in addition to trimming start and end portions of newly found matches, largely preventing the contamination with non-homologous extensions [8]. These searches were performed against two subsets of the *nr *(non-redundant) database (downloaded from the NCBI website in July 2009), containing sequences filtered by CD-HIT [54] to a maximum pairwise sequence identity of 70% and 90%. To the final multiple sequence alignment, a representation of the predicted secondary structure, generated by the *psipred *program [55], is added to improve the profile-to-profile alignment.

### Yeast mitochondrial proteomes

We collected the protein complement of the fission and budding yeast mitochondrion from the respective gene annotating consortia. Proteins with experimental evidence of mitochondrial localization were downloaded from GeneDB [56] (*S. pombe*), and the *Saccharomyces *Genome Database [57].

### Evaluation of the Ortho-Profile method with the manually curated ortholog inventory

To evaluate the quality of orthology prediction, we took orthologs of human genes that were found in both *S. pombe *and *S. cerevisiae *(356 human genes), and for which at least one of the fungal proteins was known to be mitochondrial. For every human gene, their two orthologs in fungi were compared with the fungal ortholog inventory, manually curated by the *S. pombe *community [33] (obtained on June 2009); 95%, or 337 of the fungal orthologs had the same orthologous gene in human as inferred with Ortho-Profile. Additionally, 242 human genes had orthologs in only one fungal genome (95 in *S. cerevisiae *and 147 in *S. pombe*).

### Protein domain analysis

Among orthologs determined with the Ortho-Profile method, 84% have an identical domain composition in human and fungi and for an additional 9% of orthologs (52 proteins) the human proteins contain extra domains compared to the fungal orthologs. Given the large number of proteins with identical domains, we decided to determine to what extent the domain composition data on their own can predict the subcellular localization. We found 1,627 human genes with the same PFAM [34] domain composition as yeast mitochondrial proteins (proteins without detectable domains were excluded). Of these genes, 34% (560 genes) encode proteins localized to mitochondria [11], compared to 67% for orthologs (see the Results section). This constitutes three-fold enrichment over 173 non-mitochondrial proteins, compared to 15-fold enrichment for orthologs determined by the Ortho-Profile method (see the Results section).

### Selection of COX assembly factors

We collected 42 yeast genes from databases (*Saccharomyces *Genome Database) and literature that were previously implicated in COX transcription, translation, assembly, maintenance or regulation (Table S5 in Additional file 2). Additionally, we included *YMR244C-A*, a yeast gene of unknown function that has not been previously linked to COX in yeast, but that is a paralog of the *COX12/COX6B *subunit and has a respiratory-deficient knock-out phenotype [25] (Supplemental Methods in Additional file 2).

### Integration of orthology data in the probabilistic framework

The Bayesian framework of Pagliarini and colleagues [11] integrates seven types of data (including proteomics, targeting signal prediction, presence of mitochondria-specific domain, gene expression induction upon PGC1α overexpression, and homology with yeast mitochondrial proteins) to derive high confidence mitochondrial proteins. We replaced the data on homology with yeast mitochondrial proteins by the mitochondrial protein orthology data calculated in the Ortho-Profile pipeline. The likelihood ratios of the Bayesian framework (Maestro score) [9] were updated to reflect the change, using the formula:

$${\text{L}}_{\text{orth}}={\text{log}}_{2}\left[\text{P}\left(\text{orth}|T_{\text{mito}}\right)/\text{P}\left(\text{orth}|T_{\sim \text{mito}}\right)\right]$$

where P(orth|*T*_mito_) describes the probability that the ortholog of a yeast mitochondrial protein is an experimentally confirmed mitochondrial protein in human. Analogously, P(orth|*T*_~mito_) reflects the probability that the ortholog is an experimentally confirmed non-mitochondrial human protein (based on the Gene Ontology annotation. As a result of the probabilistic integration, 15 of the 181 human proteins inferred with Ortho-Profile to be orthologous to fungal mitochondrial proteins that were previously not regarded to be mitochondrial [11] now received support from the framework at a 10% false discovery rate threshold (Table S3 in Additional file 2). Another 31 proteins of the 181 were not considered in the compendium at all. For example, PET100 and PET117 were not annotated as genes at the time when the compendium was prepared, precluding their detection in the proteomics experiment. With the inclusion of PET100 in the predicted gene set, the protein becomes identifiable in purified mitochondria.

### Cloning of the predicted COX assembly factors and plasmid construction

The predicted COX assembly factors were PCR amplified without a stop codon from a human heart cDNA library with gene-specific primers adding Attb recombination sites (see Supplemental Methods in Additional file 2 for details).

### Cell culture and transfection

T-REx™ Flp-In™ embryonic kidney 293 cells (HEK293; Invitrogen, Carlsbad, CA, USA) were maintained in DMEM (Biowhitaker, Verviers, Belgium) supplemented with 10% (v/v) fetal calf serum (FCS; PAA Laboratories, Pasching, Austria) and 1% (v/v) penicillin and streptomycin (GIBCO, Carlsbad, CA, USA), 5 μg/ml blasticidin (Invitrogen) and 300 μg/ml zeocin (Invitrogen) and grown at 37°C under an 5% CO_2 _atmosphere. For the generation of stable cell lines expressing HEK293 T-REx™ Flp-In™, cells were transfected with the GFP- and TAP-constructs together with the pOG44 recombinase expression vector using SuperFect transfection reagent (Qiagen, Hilden, Germany). Selection of stable transfectants was achieved by replacing the zeocin in the culture medium with hygromycin B (200 μg/ml; Calbiochem, Amsterdam, the Netherlands). Transgene expression was induced by adding doxycycline (Sigma, St Louis, MO, USA) to the culture medium (final concentration 1 μg/ml) for a minimum of 24 h.

### BN-PAGE, SDS-PAGE, western blotting and immunodetection

BN-PAGE was done as described before [58]. A total of 80 μg of protein was loaded per lane. Incubations with first antibodies were followed by incubations with secondary horse radish peroxidase conjugated goat-anti-mouse or goat-anti-rabbit IgGs and visualized using the enhanced chemiluminescence kit (see Supplemental Methods in Additional file 2).

### Antibodies used in BN- and SDS-PAGE analysis

Antibodies used in BN- and SDS-PAGE analysis were rabbit anti-GFP antibody (dilution 1:5,000) [59], anti-CBP antibody (dilution 1:1,000; GenScript, Piscataway, NJ, USA) anti-ND1 (dilution 1:1,000; kindly provided by A Lombès, Unite de Recherche INSERM 153, Hospital de la Salpetriere, Paris, France [60]), mouse anti-SDHA (dilution 1:10,000), anti-COX1, anti-COX2, anti-COX4, anti-ATP5A1 (dilution 1,000) and anti-Core2 (1:5,000) (all from MitoSciences, Eugene, OR, USA), anti-TOM20 antibody (dilution 1:5000; BD Transduction Laboratories, Franklin Lakes, NJ, USA) anti-CK-B 21E10 antibody (dilution 1:2,000) [61].

### Affinity purification and FT/MS analysis

T-REx™ Flp-In™ HEK293 cells were induced by doxycycline to express the TAP-tagged COX assembly factors. As a negative control unmodified HEK293 cells were used. After harvesting, cells were resuspended in lysis buffer (30 mM Tris-HCl pH 7.4, 150 mM NaCl, 1 mM EDTA, 1% (w/v) lauryl maltoside and protease inhibitor cocktail) and subjected to three cycles of freeze-thawing. The lysates were centrifuged for 10 minutes at 10,000xg after which the supernatant was incubated under rotation in the presence of Strep-tactin Superflow beads (IBA, Göttingen, Germany) for a minimum of 2 h at 4°C. After the incubation, beads were washed six times with washing buffer (30 mM Tris-HCl pH 7.4, 150 mM NaCl and 0.1% (w/v) lauryl maltoside). Retained proteins were eluted from the beads in washing buffer containing D-Desthiobiotin (IBA). Finally, the eluates were concentrated by passing them through a 3 kDa cutoff filter (Millipore, Cork, Ireland) and further processed for nanoLC-MS/MS analysis. The proteins were digested in-solution [62] and the nanoLC-MS/MS analysis was performed as described previously [63] (Supplemental Methods in Additional file 2).

### Analysis of co-purified proteins

TAP uses the InterPlay mammalian TAP-system protocol (Agilent Technologies, Santa Clara, CA, USA), which contains a streptavidin and calmodulin binding part. Mitochondrial proteins co-purified with the five TAP-tagged constructs (*C7orf44-TAP, PET100-TAP, PET117-TAP, C12orf62-TAP, AURKAIP1-TAP*) were analyzed by nanoLC-MS/MS. We selected proteins that are expressed in the transfected cells solely following the doxycycline-stimulated expression, and not detectable without the treatment (Table S4 in Additional file 2). Additionally, we removed mitochondrial proteins that are non-specifically co-purified, based on the four additional control genes encoding mitochondrial proteins that are not directly functionally linked to respiratory chain complexes (GTPBP8, C10orf65, C7orf30 and BOLA1). Proteins co-purified with these control proteins (both upon doxycycline induction, as well as in non-induced cells, 119 proteins in total) were regarded as not specific to COX maintenance and/or assembly.

### Mitochondrial translation assay

*In vivo *mitochondrial protein synthesis in cultured cells was analyzed as described previously [64]. Briefly, cells overexpressing C12orf62 and the non-induced control were labeled for 60 minutes in L-methionine and L-cysteine free DMEM containing 10% dialyzed FCS, emetine (100 ug/ml) and 200 μCi/ml [^35^S]-methionine and [^35^S]-cysteine (Tran35S-Label; MP Biomedicals, Eindhoven, The Netherlands). After labeling, cells were chased for 10 minutes in regular DMEM with 10% FCS, harvested and resuspended in PBS containing 2% (w/v) lauryl maltoside. To remove insolubilized material the lysate was centrifuged for 10 minutes at 10,000xg. Next, equal amounts of protein were separated by SDS-PAGE on a 16% gel. To visualize labeled proteins the gel was dried and exposed to a Phosphorimager screen that was subsequently scanned with a FLA5100 scanner (Fujiimager, Tilburg, the Netherlands). Equal loading of proteins was confirmed by staining the gels with Coomassie Brilliant Blue G-250 after rehydration [65].

## Abbreviations

BN: Blue Native; CBB: Coomassie Brilliant Blue G-250; CBP: calmodulin binding peptide: a part of the TAP tag; COX: cytochrome *c *oxidase; DMEM: Dulbecco's modified Eagle's medium; FCS: fetal calf serum; GFP: green fluorescent protein; HMM: hidden Markov model; LC-MS/MS: liquid chromatography tandem mass spectrometry; OXPHOS: oxidative phosphorylation; PCR: polymerase chain reaction; TAP: tandem affinity purification; TMRM: tetramethyl rhodamine methyl ester.

## Competing interests

The authors declare that they have no competing interests.

## Authors' contributions

RS conceived the study and carried out *in silico *analysis with the help of TC and EL. BW performed the experiments with the input from JE, MR, MB, JG and LH. LN and MH participated in design and coordination. RS wrote the manuscript with the input from all authors. All authors read and approved the final manuscript.

## Supplementary Material

Additional file 1 **Additional Table S6 - human orthologs of fungal mitochondrial proteins**. Click here for file

Additional file 2 **Additional Text, Tables S1 to S5 and S7 to S9 and Figures S1 to S4**. Click here for file
