# Supplementary material for: Iterative orthology prediction uncovers new mitochondrial proteins and identifies C12orf62 as the human ortholog of COX14, a protein involved in the assembly of cytochrome c oxidase
Source: Genome Biol. 2012 Feb 22;13(2):R12. doi: 10.1186/gb-2012-13-2-r12 (PMC3334569; doi:10.1186/gb-2012-13-2-r12)
Supplement: Additional file 2 — Additional Text, Tables S1 to S5 and S7 to S9 and Figures S1 to S4. [file gb-2012-13-2-r12-S2.DOC]

# Supplemental Methods

*PET100 and cytochrome* c *oxidase*. Pet100p is an inner membrane protein that is a COX assembly factor in yeast interacting with partially formed subcomplex in *S. cerevisiae* [1,2]. The human ortholog, hypothetical gene *LOC100131801* (*PET100*), spans four exons at 19p13.2 (positions chr19:7694671-7696510, the human genome assembly hg19). It contains a transmembrane region bound by perfectly conserved glutamic acid and asparagine amino acids. The amino acid conservation pattern also suggests the functional importance of the P25 residue, unusual for transmembrane regions are generally not constrained in their amino acid sequence. In yeast, *PET100* associates with COX subassembly composed of subunits VII, VIIa, and VIII and the interaction takes place in the inner membrane [2]. We purified subunit VIIa (encoded by *COX7A2* gene) using TAP-tagged *PET100* (Table 3), corroborating the interaction in human. The location of the transmembrane region (Figure S1g) is consistent with the fungal ortholog and with the membrane localization in human (Figure S2), although we cannot exclude the possibility of an ER localization (Figure S2a).

*Differences in the biogenesis of COX between mammals and yeast.* In contrast to human, the expression of COX proteins in yeast involves splicing and UTR (untranslated region)-bound factors. A number of splicing factors have been identified. Yeast mitochondrial splicing system genes (*MSS18, MSS116, PET54*) are needed for *COX1* expression and splicing in *S. cerevisiae* [3–6]. The human mitochondrial genome lacks introns and, consistently, orthologs of these splicing genes cannot be found in the human genome.

Yeast activates expression of *COX3* via its 5’ UTR-binding *PET54*, *PET122* and *PET494* [7] and expression of *COX1* via UTR-binding MSS51 [8]. The MSS51 ortholog (ZMYND17) is the only gene from the list of hypothetical assembly factors that is not supported by evidence of COX association, co-expression, or mitochondrial localization. Furthermore, ZMYND17 contains a MYND-type zinc finger domain that is conserved among its orthologs in vertebrates but absent from MSS51, suggesting a different molecular function of the proteins. We did not identify orthologs of the other three UTR-binding fungal genes, which is compatible with the lack of UTRs in mammalian mitochondria. Thus the dissimilarities in COX biogenesis between yeast and human are reflected in the absence of mitochondrial proteins specific to COX splicing and UTR-initiated translation in human.

*FT/MS analysis.* Proteins were digested in-solution as described previously, with minor modifications [9]. Briefly, samples were 1:1 (v/v) diluted with 8 M urea in 10 mM Tris pH 8 (final volume <100 µl). Proteins were reduced and alkylated with 1 mM dithiothreitol and 1 mM iodoacetamide for 1 hour in two successive steps at room temperature (RT). A two-step enzymatic digestion was performed. First, proteins were digested by addition of 1 µg lysyl endopeptidase (Lys-C) (WAKO Chemicals, San Diego, CA, USA) for 3 hours at room temperature. After dilution of the sample with 50 mM ammonium bicarbonate to a final concentration of 2 M urea, proteins were further digested overnight at 37°C by 1 µg trypsin (Promega, Madison, WI, USA). Reactions were terminated by adding trifluoroacetic acid to a final concentration of 1%. Samples were desalted and concentrated using Stage tips [10]. Nano-LC (liquid chromatography)-MS/MS (mass spectrometry) analysis was performed as described previously [11]. Chromatographic separations were performed using an in-house packed reversed-phase C18 column on an Agilent nanoflow 1100 liquid chromatograph. The nanoLC was coupled on-line via a nano-electrospray ion source (Thermo Fisher Scientific) to a 7T linear ion trap Fourier transform ion cyclotron resonance mass spectrometer (LTQ FT, Thermo Fisher Scientific, see Table S1 for instrument settings). Mass spectrometric data files were searched using the database search program Mascot (Matrix Science Inc., USA, version 2.2). The database used for the searches consisted of the human RefSeq database (release 33) with addition of the sequences of the TAP proteins used in this study and known contaminants such as trypsin and Lys-C. An in-house developed script was used to parse first-ranked peptides from the Mascot output files, and evaluate peptide and protein identifications (Table S2)

*Cloning of the predicted COX assembly factors and plasmid construction*. The predicted COX assembly factors were polymerase chain reaction (PCR) amplified without stop codon from a human heart cDNA library with gene specific primers adding Attb recombination sites (underlined) at the 5’- and 3’-end of the PCR product. The following primers were used:

C7orf44 forward: 5’-AAAAAGCAGGCTTCGCCACCatgatgtggcaaaagtatgcagg-3’.

C7orf44 reversed: 5’-AGAAAGCTGGGTGctcctttttcacttcatcaccg-3’.

PET100 forward 5’AAAAAGCAGGCTTCGCCACCatgggggtgaagctggagatatttcgg-3’.

PET100 reversed: 5’-AGAAAGCTGGGTGggagttctgctgggcgtc-3’.

PET117 forward 5’-AAAAAGCAGGCTTCGCCACCatgtctaggagctcgaaggtgg-3’.

PET117 reversed: 5’- AGAAAGCTGGGTGtgatttttgagatccttttgcc-3’.

C12orf62 forward: 5’-AAAAAGCAGGCTTCGCCACCatgccaactggcaagcagctagc-3’.

C12orf62 reversed: 5’- AGAAAGCTGGGTGcatgattcctgaggtcttc-3’.

AURKAIP1 forward: 5’- AAAAAGCAGGCTTCGCCACCatgctcctggggcgcctgacttc-3’.

AURKAIP1 reversed: 5’-AGAAAGCTGGGTGtttgccccgcaggtagatc-3’.

Next, specific PCR products were subjected to a second round of PCR with adaptor primers (forward: 5’–GGGGACAAGTTTGTACAAAAAAGCAGGCT-3’ and reversed: 5’–GGGGACCACTTTGTACAAGAAAGCTGGGT-3’) completing the Attb recombination sites, enabling the PCR products to be recombined in the pDONR210 vector using the Gateway BP Clonase II enzyme mix (Invitrogen, Carlsbad, CA, USA). With the Gateway LR clonase II enzyme mix (Invitrogen) the inserts in the pDONR201 vector were recombined in mammalian expression vectors adding a green fluorescent protein (GFP) or affinity purification (TAP) tag to the C-terminal part of the protein. Constructs were verified by sequence analysis.

*Live cell Fluorescence Microscopy imaging of TMRM.* HEK293 expressing the predicted COX assembly proteins fused to GFP were seeded on Willco dishes (Intracel Ltd., Royston, UK) and transgene expression was induced by adding doxycyline to the culture medium. When cells had reached ∼70% confluency the culture medium was replaced with medium containing 100 nM tetramethyl rhodamine methyl ester (TMRM; Invitrogen) and incubated for 25 min. at 37°C. After loading, cells were washed with phosphate buffered saline (PBS), and coverslips were mounted in a temperature-controlled chamber attached to the stage of an inverted microscope (Axiovert 200 M, Carl Zeiss, Jena, Germany). Experiments were performed at 37°C during which cells were maintained in a HEPES-Tris (HT) solution (132 mM NaCl, 4.2 mM KCl, 1 mM MgCl_2_, 5.5 Mm D-glucose, 10 mM HEPES, 1 mM CaCl_2_, pH 7.4). TMRM was excited at 540 nm with a ZEISS LSM510 Meta confocal microscope (Carl Zeiss B.V., Sliedrecht, The Netherlands). Images were acquired using a ×63 oil immersion objective (N.A. 1.4; Carl Zeiss).

*PET100 BN-PAGE, SDS-PAGE, Western blotting and immunodetection.* Blue Native polyacrylamide gel electrophoresis (BN-PAGE) was done as described before [12]. A total of 80 µg of protein was loaded per lane. For sodium dodecyl sulfate polyacrylamide gel electrophoresis (SDS-PAGE) an equal volume of Tricine sample buffer (Biorad) containing 2% [v/v] 2-mercaptoethanol was added to the samples. Resolved proteins were transferred to polyvinylidene fluoride (PVDF) or nitrocellulose membranes by Western blotting. After blocking with 5% non-fat dry milk in PBS containing 0.1% [v/v] Tween-20 (PBST) membranes were incubated with primary antibodies. Incubations with first antibodies were followed by incubations with secondary horse radish peroxidase conjugated goat-anti-mouse or goat-anti-rabbit IgGs (dilution: 1:1,000; Invitrogen). Immunoreactive bands were visualized using the enhanced chemiluminescence kit (Thermo Scientific) and detected with the ChemiDoc XRS+ system (Biorad).

*Identification of PET100 in mouse heart mitochondria.* Raw spectrum LTQ-Orbitrap files of purified heart mitochondria taken form the study of [13] were downloaded from the ProteomeCommons Tranche network (<https://proteomecommons.org/dataset.jsp?i=66721>, mouse heart mitochondria M0606223_heartmito_geLC) and re-processed by MaxQuant [14] version 1.2.13 (http://maxquant.org/) in combination with Mascot version 2.2 (Matrix Science). Peak lists were generated using a peak reduction setting of 10 peaks per 100 Da and searched against IPI mouse version 3.68 (<ftp://ftp.ebi.ac.uk/pub/databases/IPI/>, 56,729 sequences) supplemented with the sequence of PET100, sequences of frequently observed contaminants and with reversed sequences of all entries. Mascot search parameters for protein identification specified a mass tolerance of 20 ppm for the parental peptide and 0.5 Da for fragmentation spectra and a trypsin specificity allowing up to 3 miscleaved sites. Carbamidomethylation of cysteines was specified as a fixed modification, and oxidation of methionine, deamidation of glutamine and asparagine and protein N-terminal acetylation were set as variable modifications. The required minimal peptide length was set at 6 amino acids. We accepted peptides and proteins with False Discovery Rates of 0.01 and excluded proteins detected by single peptides. PET100 is detected by three unique peptides with a sequence coverage of 39.5% (Figure S3). No peptides from calreticulin or cyclophilin B proteins (ER markers) were detected in the data.

*YMR244C-A, a putative yeast COX assembly factor and an ortholog of C1orf31*. We included *YMR244C-A*, a yeast gene of unknown function that has not been previously linked to COX in yeast, but that is a paralog of *COX12/COX6B* subunit (HMM-HMM search E-value < 8.8E-23). While no function information on this yeast gene is available, large-scale analysis indicates that the gene knock-out of the overlapping reading frame leads to respiratory-deficient phenotype [15]. The overlapping reading frame is now considered dubious, therefore it is more likely that the evolutionary conserved *YMR244C-A* is responsible for the knock-out phenotype. *C1orf31* is the human ortholog of *YMR244C-A* (Figure S1k)*.*

*Homologs of COX assembly factors in B. hominis genome.* For each putative assembly factor we collected homologs in the nr database for both human and yeast sequences using three PSI-blast iterations or until convergence. Subsequently all the collected yeast and human homologs were aligned using CLUSTAL W [16] and the HMM model was built using HMMER 3.0 (http://hmmer.org). The *B. hominis* genome [17] was translated to amino acid sequence in six possible frames. The translated genome was tested for presence of homologs using HMMER 3.0 (E<0.1, default parameters except for disabled heuristics to increase the likelihood of finding a homolog).

*Known subcellular localization in mammals*. For the mitochondrial localization in human and mouse, we took into account the Mitocarta compendium and both mouse and human GO annotations. The data on subcellular localization was obtained from GO repository (http://www.ebi.ac.uk/QuickGO) on 9 June 2011. Only experimental or manually curated GO evidence codes were taken into account: IDA (inferred from direct assay), EXP (inferred from experiment), TAS (traceable author statement). All terms that are annotated with terms describing subcellular compartments (or their descendants) are taken into account. The human-mouse orthologs were extracted from the NCBI Homologene database [18]. The list of subcellular compartments of human cell is shown in the Table S8.

*D. melanogaster ortholog of PET100*. The fruit fly gene CG14483 is an ortholog of PET100 (Figure S1g). The gene highly co-expresses with subunits of respiratory chain complexes in flies (6/10 and 18/50 top co-expressing genes are OXPHOS subunits, data from STRING 9.0 [19]). The co-expression list includes COX subunits, as well as a fruit fly ortholog of PET191/C2orf64 COX assembly factor [20].

*Mitochondrial fractionation and Proteinase K protection assay*. HEK293 cell expressing PET100-GFP were fractionated as described before [21]. Equal amounts of the obtained cytoplasmic and mitochondrial fractions were analyzed with sodium dodecyl sulfate polyacrylamide gel electrophoresis (SDS-PAGE) and Western blotting. Freshly isolated mitochondria (25 µg) were treated with proteinase K, final concentrations 0.1 and 1.0 mg/ml in the absence or presence of 1% [v/v] membrane soluabilizing Triton X-100 for 15’ on ice. Reactions were stopped by the addition of phenylmethylsulfonyl fluoride (final concentration 10 mM). Susceptibility of proteins to degradation was analyzed with SDS-PAGE and Western blotting. Protein concentrations were determined using the micro BCA kit (Thermo Scientific).

**Figure S1.** A multiple sequence CLUSTAL-W alignment [16] of orthologs of fungal COX assembly proteins (a-k, Table 2). The transmembrane regions, predicted by TMHMM [22] from the human sequence, are marked above the alignment.For the alignment figures i, h, k, and j unaligned and highly divergent C-terminal regions are omitted for the clarity. The default CLUSTAL color-code was used with Jalview program to visualize the alignments [23].

a) FAM36A


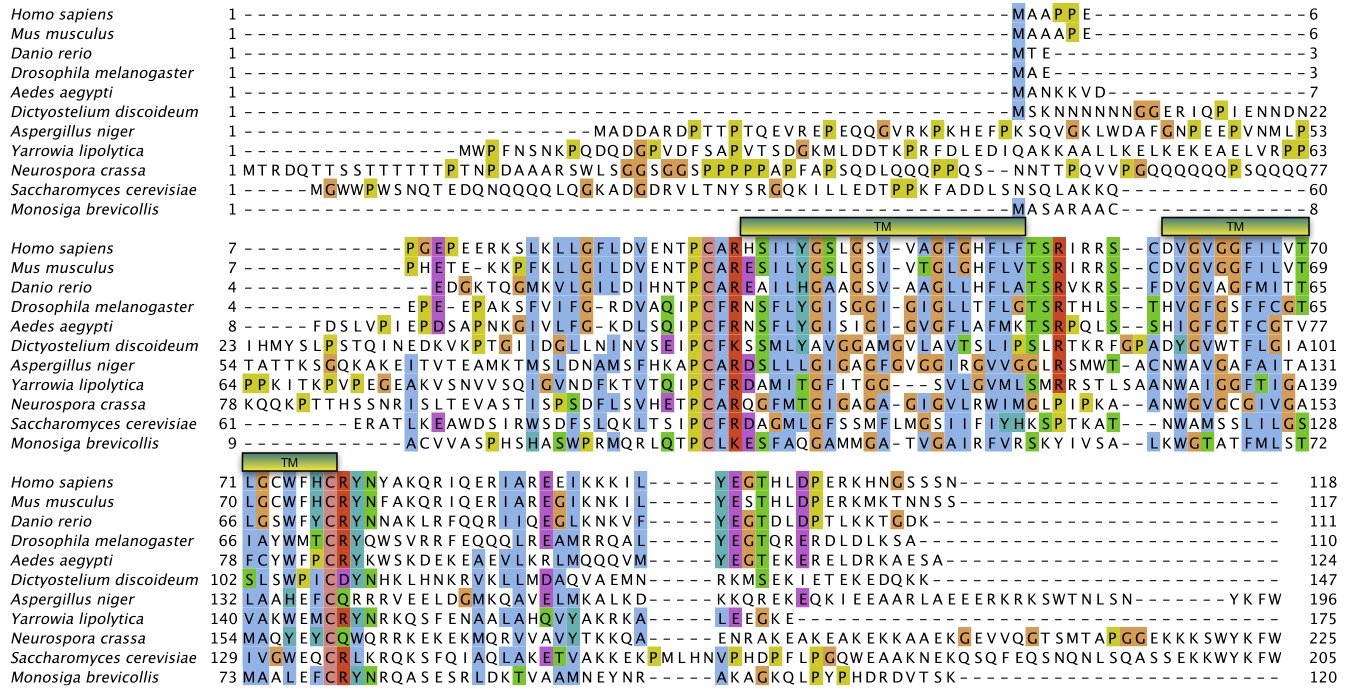


b) CHCHD7


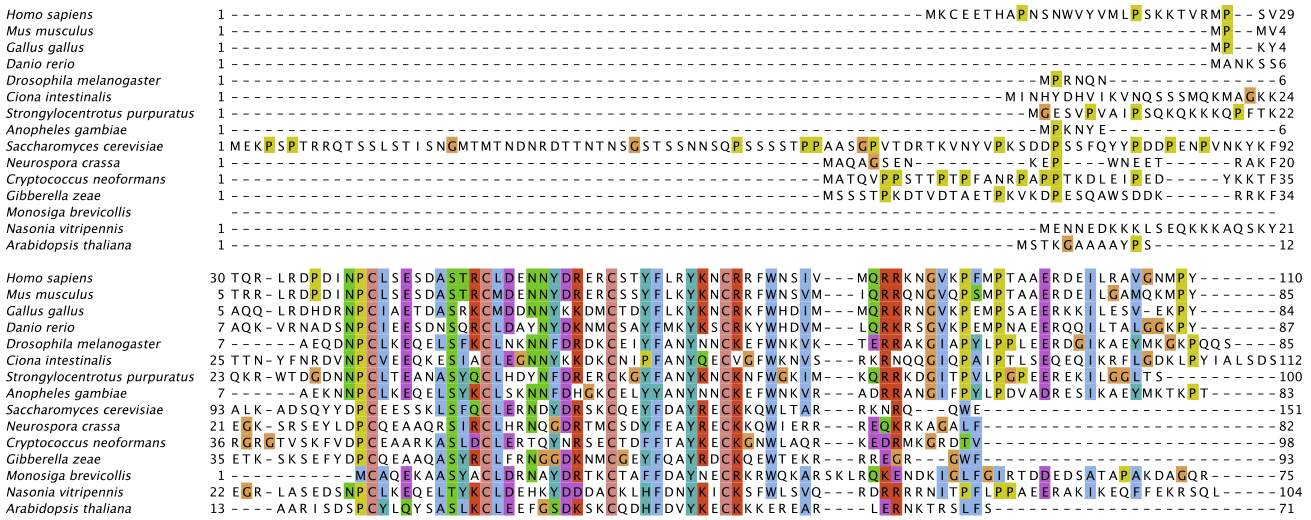


c) AURKAIP1


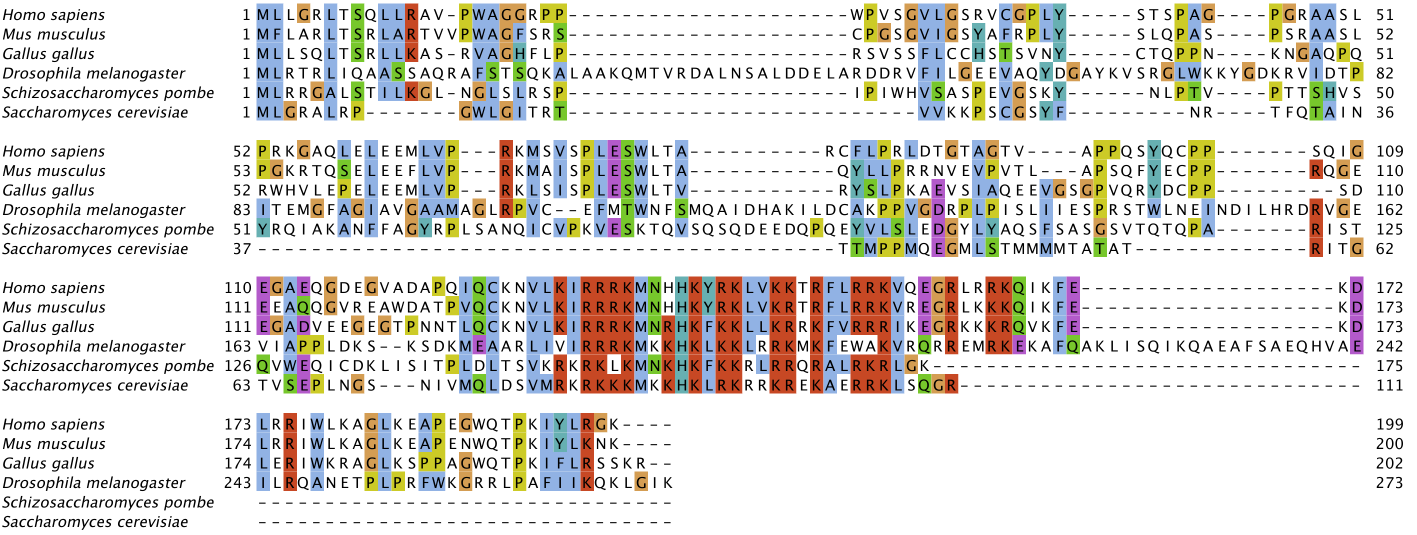


d) C7orf44


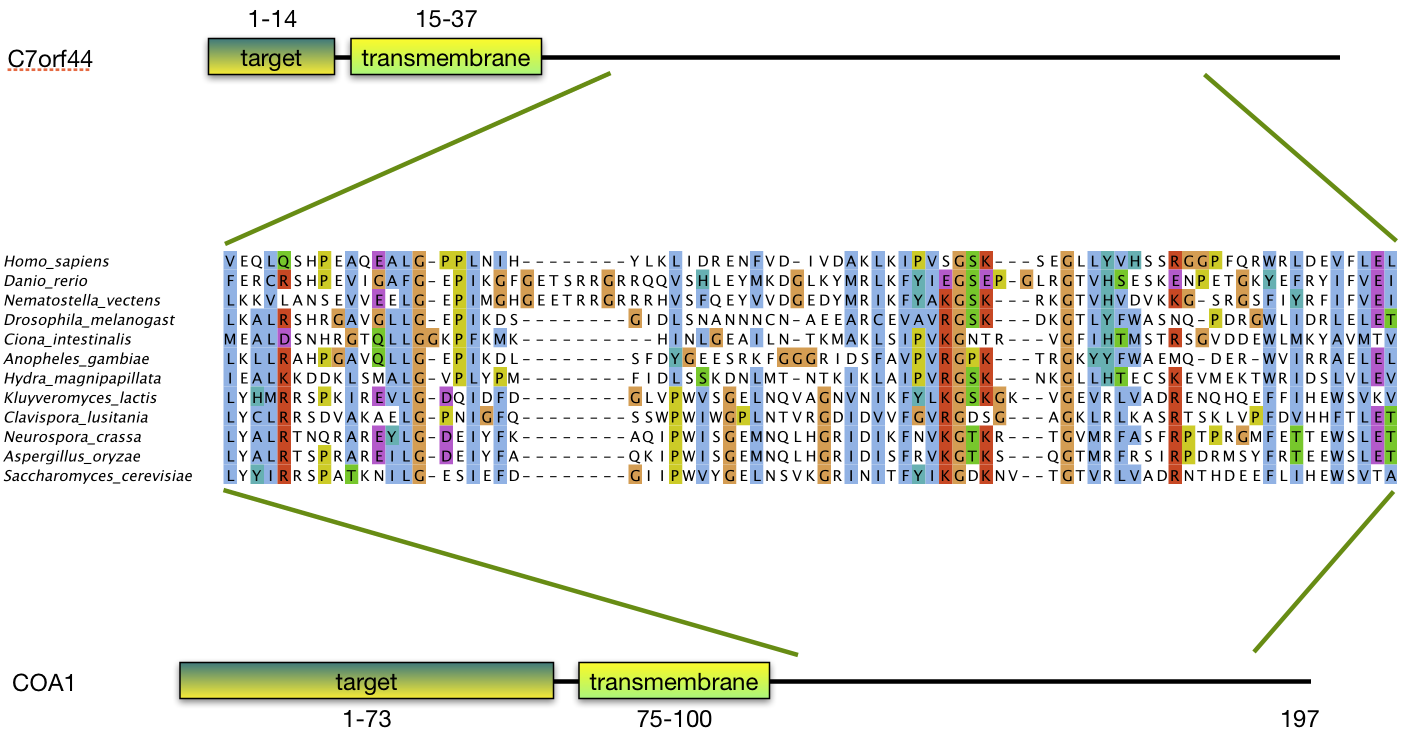


e) CCDC56


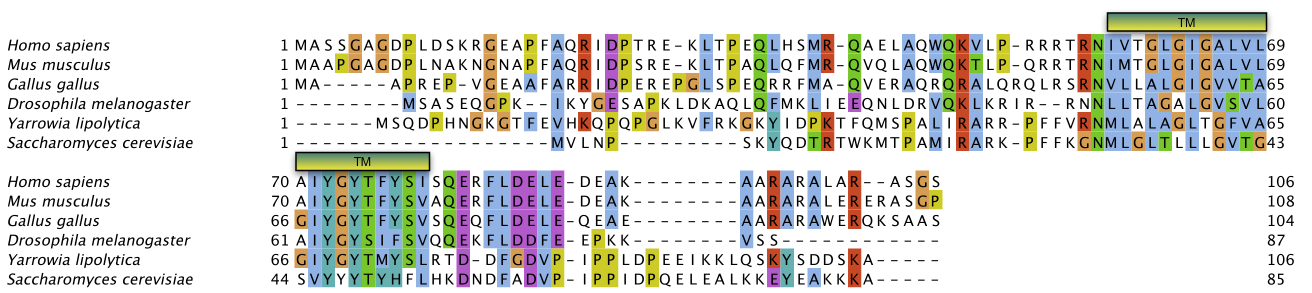


f) ZMYND17


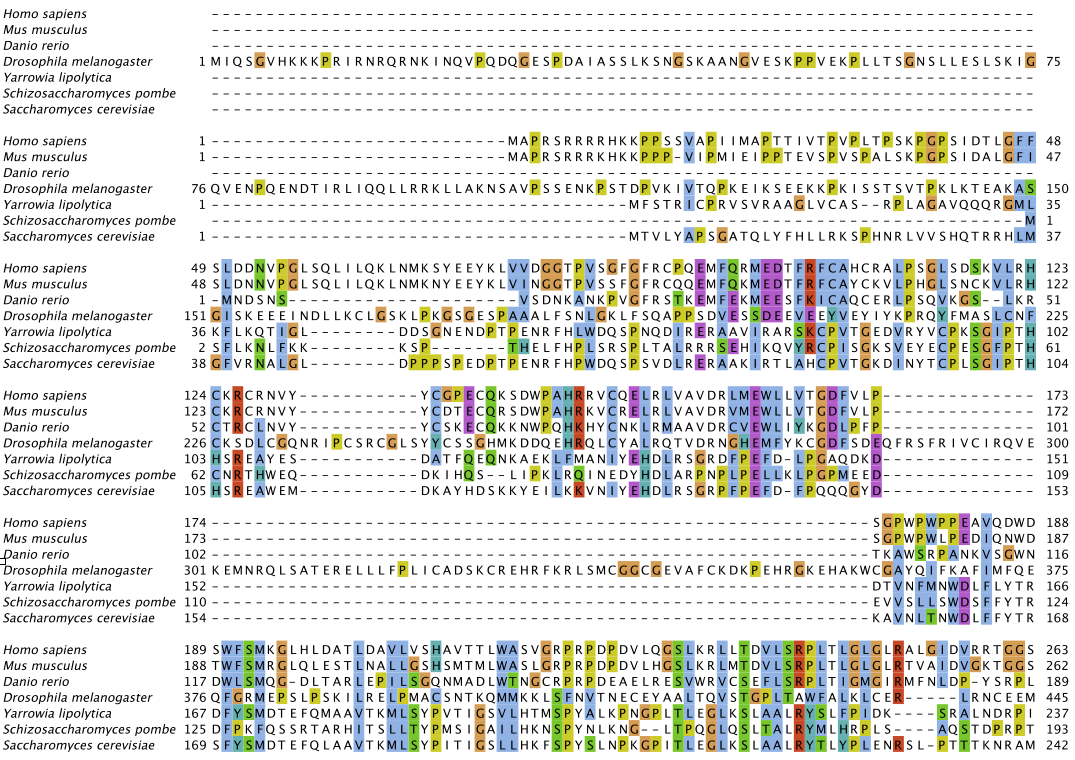


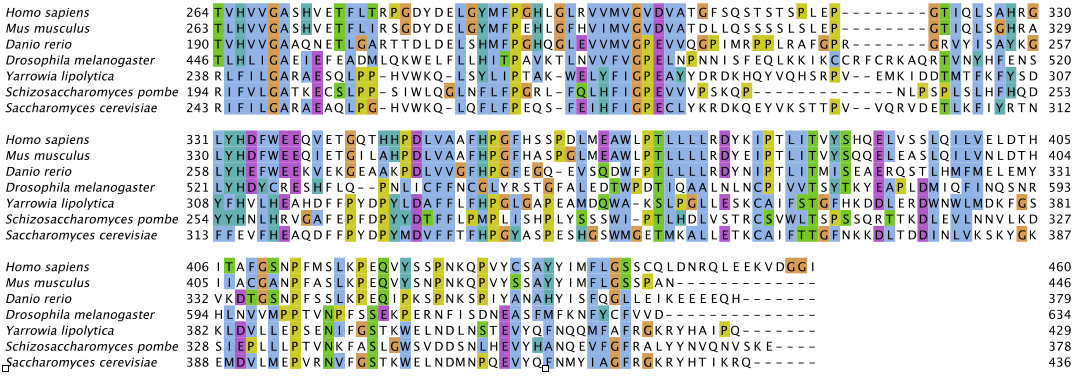


g) PET100


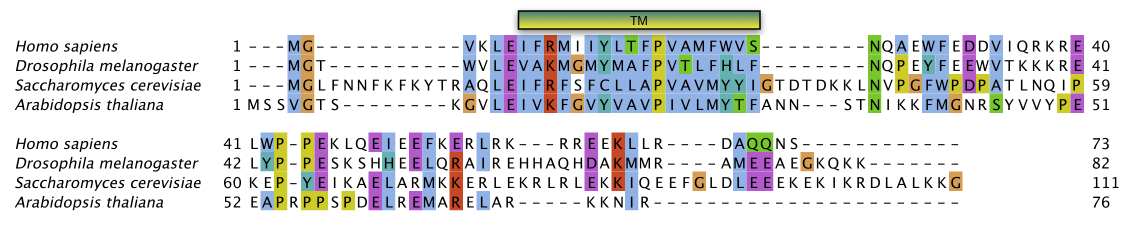


h) PET117


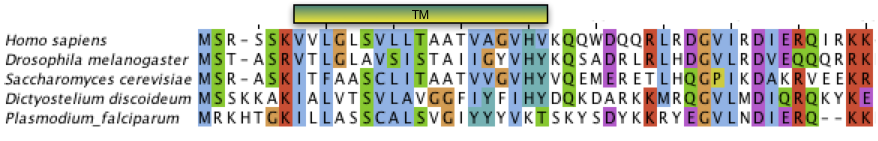


i) C2orf64


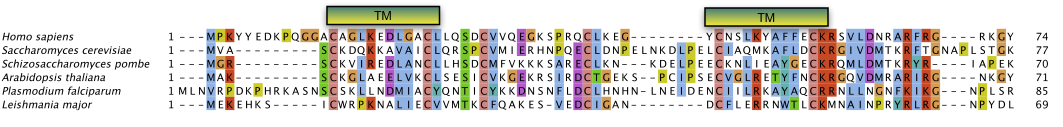


j) PTCD1


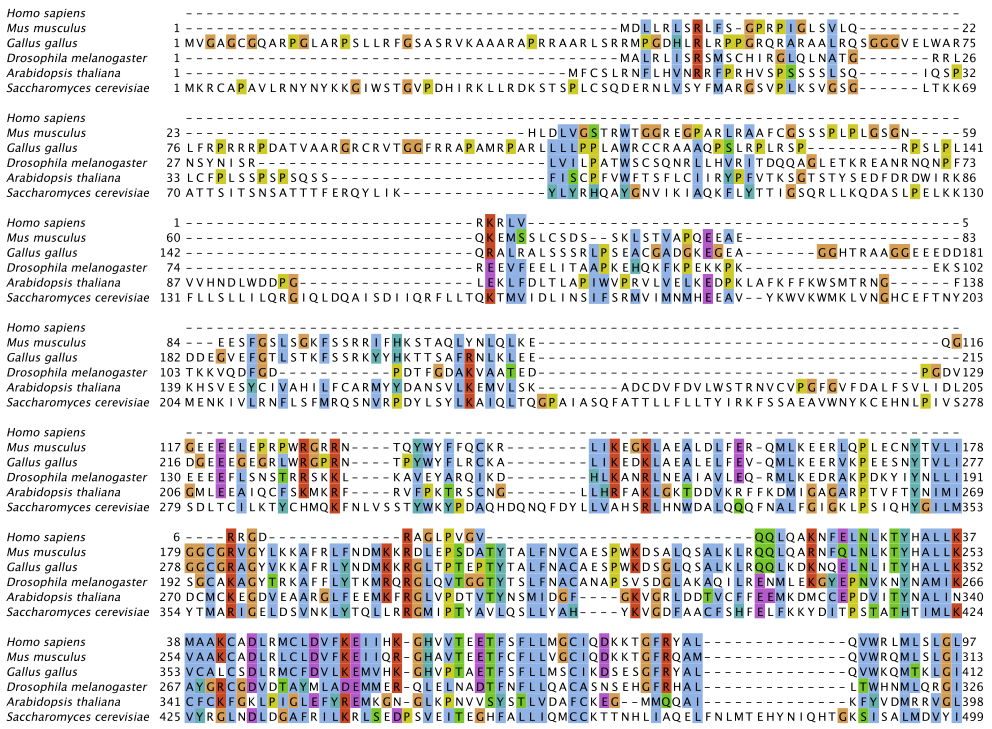


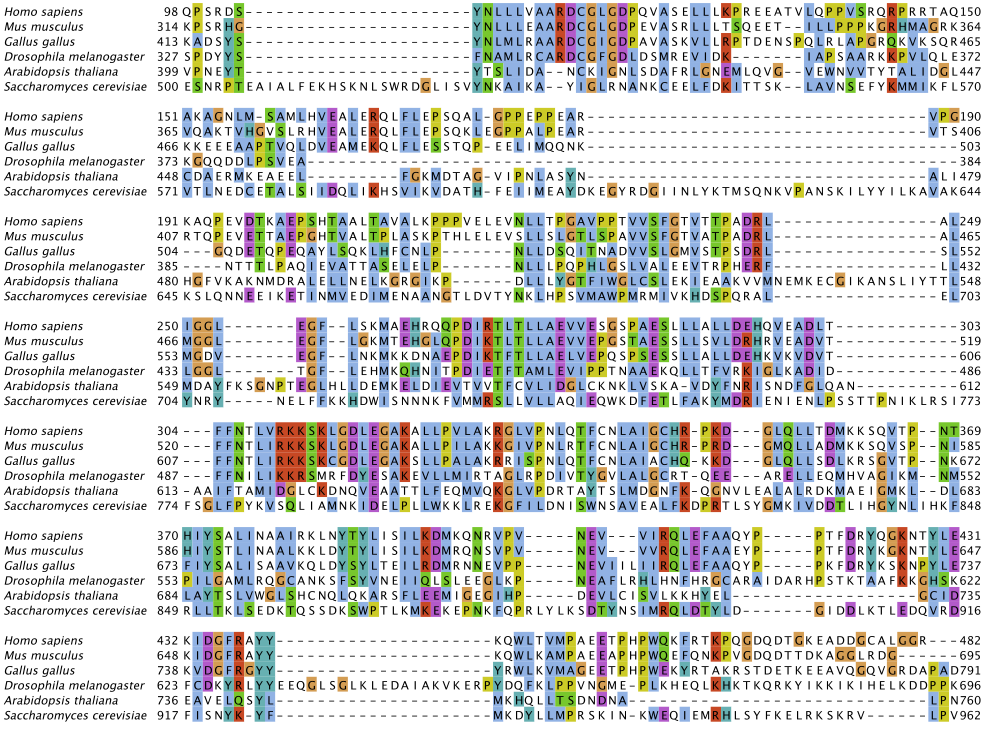


k) C1orf31


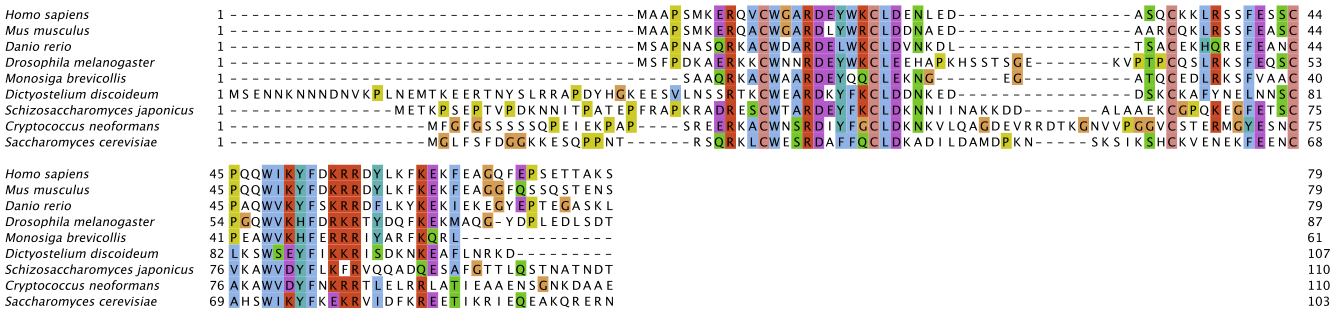


**Figure S2.** The human gene PET100 localizes to a membrane-bound organelle. a) Mitochondrial fractionation of HEK293 cells expressing PET100-GFP. SDS-PAGE analysis of obtained cell fractions probed with GFP antibody (PET100). As control of mitochondrial TOM20 (outer membrane), anti-CK-B (Creatine kinase B-type, cytoplasmic) and cyclophilin B (ER) antibodies were used. b) PET100-GFP resides inside a membrane-bound organelle. PET100-GFP is resistant to proteinase K digestion, unlike rapidly degraded TOM20 that is localized in the outer mitochondrial membrane, and matching a matrix-localized SDHA protein. Application of Triton X-100 dissolves membranes making proteins susceptible to digestion. TC - total cell lysate. Cyt - cytoplasmic and Mit - mitochondrial fractions.

**
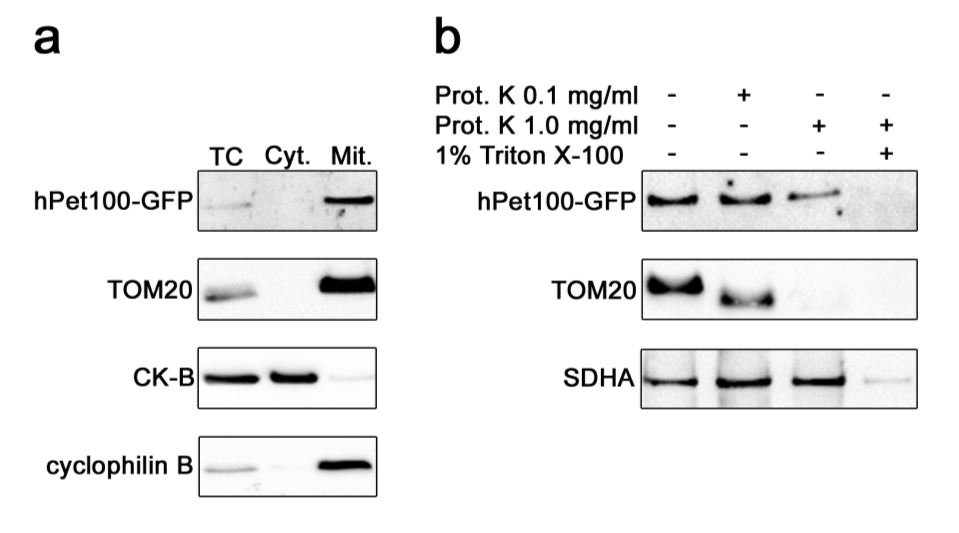
**

**Figure S3.** Mouse PET100 sequence and peptides detected in mouse mitochondria.

MGVKLEVFRMSLYLTFPVVMFWISNQAEWFEDYVVQRKRELWPR**EKEGQRQELEEFKQK**I

**RKQKEERLLQAAQQSS**

| **Sequence** | **Charge** | **m/z** | **Error [ppm]** | **Mascot Score** |
| --- | --- | --- | --- | --- |
| EGQRQELEEFKQK | 2 | 824.9181 | -0.6 | 37.47 |
| EKEGQRQELEEFKQK | 2 | 953.4869 | -0.48 | 15.46 |
| KQKEERLLQAAQQSS | 2 | 872.471 | -1.01 | 54.17 |

**Figure S4.** Fraction of human mitochondrial proteins that are homologs of yeast mitochondrial proteins. Only proteins with annotated (known) subcellular localization were considered. Independent of sequence divergence, one-to-one orthologs show much higher localization conservation compared to remaining (not one-to-one) homologs. Data from Table 1 and Table S7, the statistical significance was calculated using two-sided Fisher exact test (P<0.01, indicated by asterisk).

**
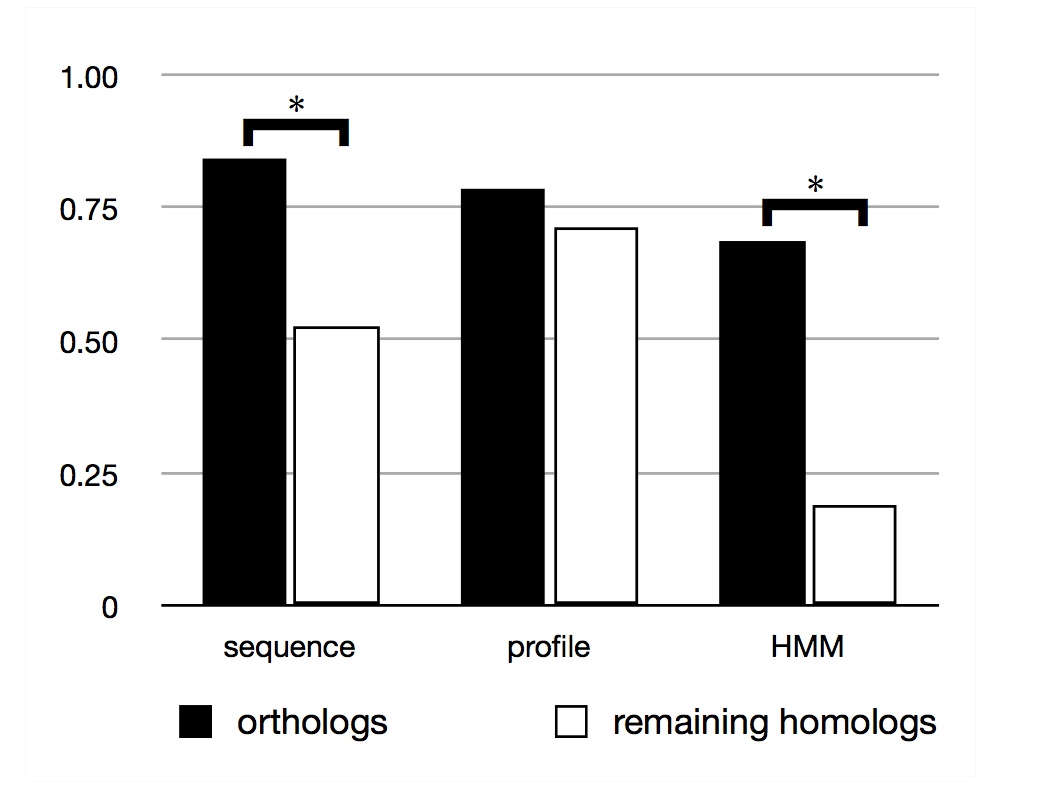
**

**Table S1: Measurement settings used in nanoLC-MS/MS analysis of TAP purified protein lysates.**

| **NanoLC Settings** | |
| --- | --- |
| Column | 15 cm fused silica emitter (New Objective, Tip: 8 +/- 1 µm, ID: 100 µm) packedwith 3 µm Reprosil C18 beads (Dr. Maisch) |
| LC method | Gradient (total 67 minutes):   - From 3% to 13% buffer B in 0.9 minutes - From 13% to 45% buffer B in 101 minutes - From 45% to 100% buffer B in 5 minutes   Column washing and equilibration:   - 100% buffer B for 20 minutes - 3% buffer B for 17 minutes |
| Buffer A | 0.5% acetic acid |
| Buffer B | 0.5% acetic acid in 100% acetonitrile |
| Flowrate | During sample loading, washing and equilibration steps: 600 nl min-1  During gradient: 300 nl min-1 |
| **MS settings** | |
| Data-dependent mode | Sequencing of the four most abundant ions |
| Dynamic exclusion | 180 s |
| Mass range FT ICR MS | 350-1,600 m/z |
| Resolution FT ICR MS | 100,000 |
| Charge state rejection | 1+ and unassigned charge states |

**Table S2.** Settings for protein identification via database searching and criteria used for validation of the results of the database searches. *) The modified delta score is derived from the Mascot delta score and denotes the difference in score between the first-ranked peptide hit and the next peptide hit with a different sequence compared to the first-ranked peptide hit.

| **Database search settings** | |
| --- | --- |
| Database |  |
| Enzymatic cleavage | Trypsin, 1 miscleavage allowed |
| Precursor ion tolerance | 10 ppm |
| Fragment ion tolerance | 0.5 Da |
| Fixed modification | Carbamidomethylation (C) |
| Variable modifications | Oxidation (M) |
| **Validation criteria** | |
| Single peptide hits | Mascot peptide score ≥ 50  Modified delta score* ≥ 10  Expectation value < 0.01  Number of variable modifications ≤ 3 |
| Proteins identified > 1 peptide | Mascot peptide score ≥ 30  Expectation value < 0.05  Number of variable modifications ≤ 5 |

**Table S3.** Bayesian integration of the fungal orthologs (this work) with the large-scale genomics data from [13] derives mitochondrial candidate proteins that are not part of Mitocarta compendium. From the list of 29 candidates proteins that have literature-supported small-scale experimental mitochondrial (6) and non-mitochondrial (8) localization were excluded, the final list of 15 proteins presented below. Proteomics evidence indicates MS/MS abundance (coverage) and enrichment in subtractive proteomics (either pure-mitochondria enriched, crude-enriched, or ambiguous) as determined in [13]. Proteins CHCHD2, SLC25A38 and NIT2 have been implicated in mitochondrial function [24–26], but the subcellular localization has yet to be experimentally confirmed on per-protein basis.

| Ensembl ID | Gene symbol | Proteomics | Description |
| --- | --- | --- | --- |
| ENSG00000144659 | *SLC25A38* |  | solute carrier family 25, member 38 |
| ENSG00000169762 | *TAPT1* |  | Transmembrane anterior posterior transformation protein 1 homolog |
| ENSG00000108602 | *ALDH3A1* | 0-25ambig | aldehyde dehydrogenase 3 family, memberA1 |
| ENSG00000189283 | *FHIT* | 50-75pure | fragile histidine triad gene |
| ENSG00000106153 | *CHCHD2* |  | coiled-coil-helix-coiled-coil-helix domain containing 2 |
| ENSG00000166133 | *RPUSD2* |  | RNA pseudouridylate synthase domain containing 2 |
| ENSG00000142657 | *PGD* | 25-50crude | phosphogluconate dehydrogenase |
| ENSG00000163170 | *BOLA3* | 50-75ambig | bolA homolog 3 (E. coli) |
| ENSG00000146151 | *HMGCLL1* | 0-25ambig | 3-hydroxymethyl-3-methylglutaryl-Coenzyme A lyase-like 1 |
| ENSG00000114021 | *NIT2* | 50-75ambig | nitrilase family, member 2 |
| ENSG00000103121 | *C16orf61* | 50-75ambig | chromosome 16 open reading frame 61 |
| ENSG00000189201 | *COX19* | 0-25ambig | COX19 cytochrome c oxidase assembly homolog (S. cerevisiae) |
| ENSG00000185633 | *NDUFA4L2* | 25-50ambig | NADH dehydrogenase (ubiquinone) 1 alpha subcomplex, 4-like 2 |
| ENSG00000183978 | *CCDC56* | 25-50pure | coiled-coil domain containing 56 |
| ENSG00000131069 | *ACSS2* |  | acyl-CoA synthetase short-chain family member 2 |

**Table S4.** Mitochondrial proteins purified using NanoLC-MS/MS. Tap-tagged proteins are marked in color. COX-associated proteins (COX17, C1orf31, COX7A2) are marked bold. Listed (with the corresponding exponentially modified protein abundance index, or *emPAI* [27]) are co-purified proteins that were detectable only upon doxycycline-stimulated expression (see Methods).

| **PROTEIN** | **emPAI** |
| --- | --- |
| gi|99999997|ref|NP_9999999.7| AKIP_HUMAN Aurora kinase A-interacting protein + STREP-CAL tag [Homo sapiens] | 3.328761281 |
| gi|22547114|ref|NP_660298.2| mitochondrial ribosomal protein L10 precursor [Homo sapiens] | 0.211527659 |
| gi|4503685|ref|NP_001995.1| farnesyl diphosphate synthase isoform a [Homo sapiens] | 0.14504757 |
| gi|16905526|ref|NP_387506.1| death-associated protein 3 [Homo sapiens] | 0.115883993 |
| gi|25777734|ref|NP_003739.2| aldehyde dehydrogenase 4A1 precursor [Homo sapiens] | 0.085711119 |
| gi|7657581|ref|NP_055066.1| solute carrier family 25, member 13 (citrin) [Homo sapiens] | 0.079775162 |
| gi|4758332|ref|NP_004449.1| acyl-CoA synthetase long-chain family member 4 isoform 1 [Homo sapiens] | 0.077105056 |
| gi|188497754|ref|NP_000179.2| hexokinase 1 isoform HKI [Homo sapiens] | 0.051330177 |
|  |  |
| **PROTEIN** | **emPAI** |
| gi|99999994|ref|NP_9999999.4|CL062_HUMAN Uncharacterized protein C12orf62 + STREP-CAL tag [Homo sapiens] | 3.216965034 |
| gi|18152785|ref|NP_542786.1| reactive oxygen species modulator 1 [Homo sapiens] | 1.15443469 |
| **gi|5031645|ref|NP_005685.1| COX17 homolog, cytochrome c oxidase assembly protein [Homo sapiens]** | **1.15443469** |
| gi|51479137|ref|NP_001003696.1| ATP synthase, H+ transporting, mitochondrial F0 complex, subunit F6 isoform a precursor [Homo sapiens] | 0.467799268 |
| gi|4758984|ref|NP_004654.1| Ras-related protein Rab-11A [Homo sapiens] | 0.42510267 |
| gi|7330335|ref|NP_039234.1| chloride intracellular channel 4 [Homo sapiens] | 0.359356391 |
| gi|4758762|ref|NP_004530.1| asparaginyl-tRNA synthetase [Homo sapiens] | 0.221677349 |
| gi|22547114|ref|NP_660298.2| mitochondrial ribosomal protein L10 precursor [Homo sapiens] | 0.211527659 |
| gi|7661646|ref|NP_056338.1| small fragment nuclease [Homo sapiens] | 0.193776642 |
| gi|194097323|ref|NP_004083.3| mitochondrial short-chain enoyl-coenzyme A hydratase 1 precursor [Homo sapiens] | 0.165914401 |
| gi|217330598|ref|NP_057164.3| glyoxalase domain containing 4 [Homo sapiens] | 0.14504757 |
| gi|50592988|ref|NP_003357.2| ubiquinol-cytochrome c reductase core protein II [Homo sapiens] | 0.14504757 |
| gi|52630440|ref|NP_036313.3| FK506-binding protein 8 [Homo sapiens] | 0.14504757 |
| gi|4503685|ref|NP_001995.1| farnesyl diphosphate synthase isoform a [Homo sapiens] | 0.14504757 |
| gi|4501867|ref|NP_001089.1| aconitase 2 precursor [Homo sapiens] | 0.140624924 |
| gi|62420877|ref|NP_001014763.1| electron-transfer-flavoprotein, beta polypeptide isoform 2 [Homo sapiens] | 0.136463666 |
| gi|183227688|ref|NP_001116850.1| adenylosuccinate lyase isoform b [Homo sapiens] | 0.128837892 |
| gi|6681764|ref|NP_004993.1| NADH dehydrogenase (ubiquinone) 1 alpha subcomplex, 9, 39kDa [Homo sapiens] | 0.110336318 |
| gi|73466520|ref|NP_001026976.1| aldehyde dehydrogenase 3A2 isoform 1 [Homo sapiens] | 0.110336318 |
| gi|24308013|ref|NP_055975.1| peptidase (mitochondrial processing) alpha [Homo sapiens] | 0.089022962 |
| gi|75677353|ref|NP_114127.3| AAA-ATPase TOB3 [Homo sapiens] | 0.082636734 |
| gi|7657581|ref|NP_055066.1| solute carrier family 25, member 13 (citrin) [Homo sapiens] | 0.079775162 |
| gi|4885281|ref|NP_005262.1| glutamate dehydrogenase 1 [Homo sapiens] | 0.079775162 |
| gi|42490749|ref|NP_004290.2| ATP-binding cassette, sub-family B, member 7 [Homo sapiens] | 0.077105056 |
| gi|4505145|ref|NP_002387.1| malic enzyme 2, NAD(+)-dependent, mitochondrial [Homo sapiens] | 0.077105056 |
| gi|4504505|ref|NP_000405.1| hydroxysteroid (17-beta) dehydrogenase 4 [Homo sapiens] | 0.077105056 |
| gi|41393561|ref|NP_056991.2| leucine aminopeptidase 3 [Homo sapiens] | 0.072267222 |
| gi|145553959|ref|NP_060597.4| elaC homolog 2 [Homo sapiens] | 0.060818355 |
|  |  |
| **PROTEIN** | **emPAI** |
| gi|99999998|ref|NP_9999999.8| CG044_HUMAN Uncharacterized protein C7orf44 + STREP-CAL tag [Homo sapiens] | 2.16227766 |
| **gi|5031645|ref|NP_005685.1| COX17 homolog, cytochrome c oxidase assembly protein [Homo sapiens]** | **1.15443469** |
| gi|51479139|ref|NP_001003697.1| ATP synthase, H+ transporting, mitochondrial F0 complex, subunit F6 isoform a precursor [Homo sapiens] | 0.467799268 |
| **gi|61175258|ref|NP_001013003.1| C1orf31, hypothetical protein LOC388753 [Homo sapiens]** | **0.467799268** |
| gi|194097323|ref|NP_004083.3| mitochondrial short-chain enoyl-coenzyme A hydratase 1 precursor [Homo sapiens] | 0.359356391 |
| gi|22547114|ref|NP_660298.2| mitochondrial ribosomal protein L10 precursor [Homo sapiens] | 0.211527659 |
| gi|4758984|ref|NP_004654.1| Ras-related protein Rab-11A [Homo sapiens] | 0.193776642 |
| gi|13569962|ref|NP_112243.1| RAB1B, member RAS oncogene family [Homo sapiens] | 0.193776642 |
| gi|7330335|ref|NP_039234.1| chloride intracellular channel 4 [Homo sapiens] | 0.165914401 |
| gi|50592988|ref|NP_003357.2| ubiquinol-cytochrome c reductase core protein II [Homo sapiens] | 0.14504757 |
| gi|171906589|ref|NP_005872.2| branched chain ketoacid dehydrogenase kinase isoform a [Homo sapiens] | 0.136463666 |
| gi|112380628|ref|NP_005552.3| lysosomal-associated membrane protein 1 [Homo sapiens] | 0.128837892 |
| gi|4758762|ref|NP_004530.1| asparaginyl-tRNA synthetase [Homo sapiens] | 0.105295141 |
| gi|75677353|ref|NP_114127.3| AAA-ATPase TOB3 [Homo sapiens] | 0.082636734 |
| gi|4758332|ref|NP_004449.1| acyl-CoA synthetase long-chain family member 4 isoform 1 [Homo sapiens] | 0.077105056 |
| gi|4504505|ref|NP_000405.1| hydroxysteroid (17-beta) dehydrogenase 4 [Homo sapiens] | 0.077105056 |
| gi|194272210|ref|NP_001123561.1| lysyl-tRNA synthetase isoform 1 [Homo sapiens] | 0.077105056 |
| gi|4501867|ref|NP_001089.1| aconitase 2 precursor [Homo sapiens] | 0.068000433 |
| gi|14043022|ref|NP_004981.2| methionyl-tRNA synthetase [Homo sapiens] | 0.064209244 |
| gi|188497754|ref|NP_000179.2| hexokinase 1 isoform HKI [Homo sapiens] | 0.051330177 |
|  |  |
| **PROTEIN** | **emPAI** |
| gi|99999996|ref|NP_9999999.6| PET100_Human + STREP-CAL tag [Homo sapiens] | 2.16227766 |
| **gi|5031645|ref|NP_005685.1| COX17 homolog, cytochrome c oxidase assembly protein [Homo sapiens]** | **1.15443469** |
| **gi|4502989|ref|NP_001856.1| cytochrome c oxidase subunit VIIa polypeptide 2 (liver) precursor [Homo sapiens]** | **1.15443469** |
| gi|51479129|ref|NP_001003713.1| ATP synthase, H+ transporting, mitochondrial F0 complex, subunit F2 isoform 2b [Homo sapiens] | 0.77827941 |
| gi|194097323|ref|NP_004083.3| mitochondrial short-chain enoyl-coenzyme A hydratase 1 precursor [Homo sapiens] | 0.359356391 |
| gi|22547114|ref|NP_660298.2| mitochondrial ribosomal protein L10 precursor [Homo sapiens] | 0.211527659 |
| gi|4758984|ref|NP_004654.1| Ras-related protein Rab-11A [Homo sapiens] | 0.193776642 |
| gi|217330598|ref|NP_057164.3| glyoxalase domain containing 4 [Homo sapiens] | 0.14504757 |
| gi|50592988|ref|NP_003357.2| ubiquinol-cytochrome c reductase core protein II [Homo sapiens] | 0.14504757 |
| gi|4503685|ref|NP_001995.1| farnesyl diphosphate synthase isoform a [Homo sapiens] | 0.14504757 |
| gi|20127408|ref|NP_000173.2| mitochondrial trifunctional protein, alpha subunit precursor [Homo sapiens] | 0.136463666 |
| gi|171906589|ref|NP_005872.2| branched chain ketoacid dehydrogenase kinase isoform a [Homo sapiens] | 0.136463666 |
| gi|112380628|ref|NP_005552.3| lysosomal-associated membrane protein 1 [Homo sapiens] | 0.128837892 |
| gi|25777736|ref|NP_733844.1| aldehyde dehydrogenase 4A1 precursor [Homo sapiens] | 0.085711119 |
| gi|7657581|ref|NP_055066.1| solute carrier family 25, member 13 (citrin) [Homo sapiens] | 0.079775162 |
| gi|4885281|ref|NP_005262.1| glutamate dehydrogenase 1 [Homo sapiens] | 0.079775162 |
| gi|4758332|ref|NP_004449.1| acyl-CoA synthetase long-chain family member 4 isoform 1 [Homo sapiens] | 0.077105056 |
| gi|4504505|ref|NP_000405.1| hydroxysteroid (17-beta) dehydrogenase 4 [Homo sapiens] | 0.077105056 |
| gi|15991831|ref|NP_277033.1| hexokinase 1 isoform HKI-ta/tb [Homo sapiens] | 0.05021108 |
|  |  |
| **PROTEIN** | **emPAI** |
| **gi|99999995|ref|NP_001158283| PET117_Human cytochrome c oxidase assembly factor-like + STREP-CAL tag [Homo sapiens]** | **12.33521432** |
| **gi|5031645|ref|NP_005685.1| COX17 homolog, cytochrome c oxidase assembly protein [Homo sapiens]** | **1.15443469** |
| gi|116517313|ref|NP_004861.2| mannose-P-dolichol utilization defect 1 [Homo sapiens] | 0.930697729 |
| gi|51479129|ref|NP_001003713.1| ATP synthase, H+ transporting, mitochondrial F0 complex, subunit F2 isoform 2b [Homo sapiens] | 0.77827941 |
| gi|7330335|ref|NP_039234.1| chloride intracellular channel 4 [Homo sapiens] | 0.359356391 |
| gi|62530384|ref|NP_001910.2| dodecenoyl-Coenzyme A delta isomerase precursor [Homo sapiens] | 0.258925412 |
| gi|22547114|ref|NP_660298.2| mitochondrial ribosomal protein L10 precursor [Homo sapiens] | 0.211527659 |
| gi|4758984|ref|NP_004654.1| Ras-related protein Rab-11A [Homo sapiens] | 0.193776642 |
| gi|13569962|ref|NP_112243.1| RAB1B, member RAS oncogene family [Homo sapiens] | 0.193776642 |
| gi|194097323|ref|NP_004083.3| mitochondrial short-chain enoyl-coenzyme A hydratase 1 precursor [Homo sapiens] | 0.165914401 |
| gi|21361356|ref|NP_006800.2| translocase of outer mitochondrial membrane 34 [Homo sapiens] | 0.154781985 |
| gi|50592988|ref|NP_003357.2| ubiquinol-cytochrome c reductase core protein II [Homo sapiens] | 0.14504757 |
| gi|4503685|ref|NP_001995.1| farnesyl diphosphate synthase isoform a [Homo sapiens] | 0.14504757 |
| gi|73466520|ref|NP_001026976.1| aldehyde dehydrogenase 3A2 isoform 1 [Homo sapiens] | 0.110336318 |
| gi|188497754|ref|NP_000179.2| hexokinase 1 isoform HKI [Homo sapiens] | 0.105295141 |
| gi|75677353|ref|NP_114127.3| AAA-ATPase TOB3 [Homo sapiens] | 0.082636734 |
| gi|7657581|ref|NP_055066.1| solute carrier family 25, member 13 (citrin) [Homo sapiens] | 0.079775162 |
| gi|4758332|ref|NP_004449.1| acyl-CoA synthetase long-chain family member 4 isoform 1 [Homo sapiens] | 0.077105056 |
| gi|4504505|ref|NP_000405.1| hydroxysteroid (17-beta) dehydrogenase 4 [Homo sapiens] | 0.077105056 |
| gi|194272210|ref|NP_001123561.1| lysyl-tRNA synthetase isoform 1 [Homo sapiens] | 0.077105056 |
| gi|4501867|ref|NP_001089.1| aconitase 2 precursor [Homo sapiens] | 0.068000433 |

**Table S5.** List of COX assembly, translation and maintenance proteins collected from the literature, together with their human putative orthologs inferred with Ortho-Profile. The rightmost column indicates whether the human gene was implicated in COX assembly prior to this work.

|  | **Yeast gene** | **Human ortholog** | **Novel COX assembly factor candidate** |
| --- | --- | --- | --- |
|  | **Translational activators or regulators** |  |  |
| 1 | *PET309* | *PTCD1* | + |
| 2 | *MSS51* | *ZMYND17* | + |
| 3 | *COX14* | *C12orf62* | + |
| 4 | *PET111* |  |  |
| 5 | *PET122* |  |  |
| 6 | *PET494* |  |  |
| 7 | *COA3* | *CCDC56* | + |
|  | **Membrane insertion** |  |  |
| 8 | *OXA1* | *OXA1L* |  |
| 9 | *MBA1* | *MRPL45* |  |
| 10 | *COX18* |  |  |
| 11 | *MSS2* |  |  |
| 12 | *PNT1* |  |  |
|  | **Subunit-specific chaperones** |  |  |
| 13 | *COX20* | *FAM36A* | + |
|  | **Copper insertion** |  |  |
| 14 | *COX17* | *COX17* |  |
| 15 | *COX11* | *COX11* |  |
| 16 | *SCO1* | *SCO1* |  |
| 17 | *SCO2* |  |  |
|  | **Heme biosynthesis** |  |  |
| 18 | *COX10* | *COX10* |  |
| 19 | *COX15* | *COX15* |  |
|  | **Assembly chaperones** |  |  |
| 20 | *SHY1* | *SURF1* |  |
| 21 | *PET100* | *PET100 (LOC100131801)* | + |
|  | **Splicing factors** |  |  |
| 22 | *PET54* |  |  |
| 23 | *MSS18* |  |  |
| 24 | *MSS116* |  |  |
|  | **Other/unknown** |  |  |
| 25 | *COA1* | *C7orf44* | + |
| 26 | *PET117* | *PET117 (LOC100303755)* | + |
| 27 | *PET191* | *C2orf64* |  |
| 28 | *COA2* |  |  |
| 29 | *COX23* | *CHCHD7* | + |
| 30 | *YAH1* |  |  |
| 31 | *ARH1* | *FDXR* |  |
| 32 | *YFH1* | *FXN* |  |
| 33 | *SOM1* |  |  |
| 34 | *COX24* | *AURKAIP1* | + |
| 35 | *YMR244C-A* | *C1orf31* | + |
| 36 | *SSC1* | *HSPA9* |  |
| 37 | *COX19* |  |  |
| 38 | *Mia40* | *CHCHD4* |  |
| 39 | *COX16* | *COX16* |  |
| 40 | *IMP1* | *IMMP1L* |  |
| 41 | *IMP2* |  |  |
| 42 | *PET112* | *PET112* | + |

**Table S7.** The subcellular localization of human homologs (but not orthologs) of yeast mitochondrial proteins. Mitochondrial and non-mitochondrial proteins determined as described in Supplemental Methods. *) corroborated by experimental evidence independently in human and mouse.

|  | Homology | | | |
| --- | --- | --- | --- | --- |
|  | sequence | profile | HMM | total (localization) |
| mitochondrial | 20 | 5 | 13 | 38 (13*) |
| non-mitochondrial | 18 | 2 | 55 | 75 (20*) |
| unknown | 22 | 8 | 69 | 99 |
| total (method) | 60 | 15 | 137 | 212 |

**Table S8.** The list of GO categories that represent subcellular compartments of human cell.

| Category | Name |
| --- | --- |
| GO:0005830 | cytosolic ribosome |
| GO:0005768 | endosome |
| GO:0005794 | Golgi apparatus |
| GO:0005829 | cytosol |
| GO:0005792 | microsome |
| GO:0005739 | mitochondrion |
| GO:0009986 | cell surface |
| GO:0005856 | cytoskeleton |
| GO:0005923 | tight junction |
| GO:0042579 | microbody |
| GO:0005773 | vacuole |
| GO:0042470 | melanosome |
| GO:0005911 | intercellular junction |
| GO:0031012 | extracellular matrix |
| GO:0005576 | extracellular region |
| GO:0009579 | thylakoid |
| GO:0005783 | endoplasmic reticulum |
| GO:0016023 | cytoplasmic vesicle |
| GO:0005905 | coated pit |
| GO:0005886 | plasma membrane |
| GO:0000151 | ubiquitin ligase complex |
| GO:0000785 | chromatin |
| GO:0005634 | nucleus |
| GO:0009434 | flagellum (Eukaryota) |
| GO:0005929 | cilium |

**Table S9.** Relative intensities of ^35^S labeled mitochondrial translation products of doxycycline induced C12orf62-GFP expressing HEK293 cells. Intensities of non-induced cells (control) were set at 1.00.  Intensities were determined with the online available ImageJ software [28].

| Translation products | Gel 1 | Gel 2 |
| --- | --- | --- |
| COX1  Cyt *b*  ND2/ND1  COX2/COX3  ATP6 | 0.77  0.90  0.76  0.46  1.34 | 0.77  0.90  0.70  0.44  1.28 |

**Supplemental References**

1. Forsha D, Church C, Wazny P, Poyton RO: Structure and function of Pet100p, a molecular chaperone required for the assembly of cytochrome c oxidase in Saccharomyces cerevisiae. *Biochem. Soc. Trans* 2001, 29:436-441.

2. Church, Goehring B, Forsha D, Wazny P, Poyton RO: A role for Pet100p in the assembly of yeast cytochrome c oxidase: interaction with a subassembly that accumulates in a pet100 mutant. *J. Biol. Chem* 2005, 280:1854-186310.1074/jbc.M410726200.

3. Séraphin B, Simon M, Faye G: MSS18, a yeast nuclear gene involved in the splicing of intron aI5 beta of the mitochondrial cox1 transcript. *EMBO J* 1988, 7:1455-1464.

4. Huang H-R, Rowe CE, Mohr S, Jiang Y, Lambowitz AM, Perlman PS: The splicing of yeast mitochondrial group I and group II introns requires a DEAD-box protein with RNA chaperone function. *Proc. Natl. Acad. Sci. U.S.A* 2005, 102:163-16810.1073/pnas.0407896101.

5. Tavares-Carreón F, Camacho-Villasana Y, Zamudio-Ochoa A, Shingú-Vázquez M, Torres-Larios A, Pérez-Martínez X: The pentatricopeptide repeats present in Pet309 are necessary for translation but not for stability of the mitochondrial COX1 mRNA in yeast. *J. Biol. Chem* 2008, 283:1472-147910.1074/jbc.M708437200.

6. Valencik ML, Kloeckener-Gruissem B, Poyton RO, McEwen JE: Disruption of the yeast nuclear PET54 gene blocks excision of mitochondrial intron aI5 beta from pre-mRNA for cytochrome c oxidase subunit I. *EMBO J* 1989, 8:3899-3904.

7. Naithani S, Saracco SA, Butler CA, Fox TD: Interactions among COX1, COX2, and COX3 mRNA-specific translational activator proteins on the inner surface of the mitochondrial inner membrane of Saccharomyces cerevisiae. *Mol. Biol. Cell* 2003, 14:324-33310.1091/mbc.E02-08-0490.

8. Perez-Martinez X, Broadley SA, Fox TD: Mss51p promotes mitochondrial Cox1p synthesis and interacts with newly synthesized Cox1p. *EMBO J* 2003, 22:5951-596110.1093/emboj/cdg566.

9. Wessels HJCT, Gloerich J, van der Biezen E, Jetten MSM, Kartal B: Liquid chromatography-mass spectrometry-based proteomics of Nitrosomonas. *Meth. Enzymol* 2011, 486:465-48210.1016/B978-0-12-381294-0.00021-3.

10. Rappsilber J, Ishihama Y, Mann M: Stop and go extraction tips for matrix-assisted laser desorption/ionization, nanoelectrospray, and LC/MS sample pretreatment in proteomics. *Anal. Chem* 2003, 75:663-670.

11. Wessels HJCT, Vogel RO, van den Heuvel L, Smeitink JA, Rodenburg RJ, Nijtmans LG, Farhoud MH: LC-MS/MS as an alternative for SDS-PAGE in blue native analysis of protein complexes. *Proteomics* 2009, 9:4221-422810.1002/pmic.200900157.

12. Ugalde C, Vogel R, Huijbens R, Van Den Heuvel B, Smeitink J, Nijtmans L: Human mitochondrial complex I assembles through the combination of evolutionary conserved modules: a framework to interpret complex I deficiencies. *Hum. Mol. Genet* 2004, 13:2461-7215317750.

13. Pagliarini DJ, Calvo SE, Chang B, Sheth SA, Vafai SB, Ong S-E, Walford GA, Sugiana C, Boneh A, Chen WK, Hill DE, Vidal M, Evans JG, Thorburn DR, Carr SA, Mootha VK: A mitochondrial protein compendium elucidates complex I disease biology. *Cell* 2008, 134:112-23S0092-8674(08)00768-X.

14. Cox J, Mann M: MaxQuant enables high peptide identification rates, individualized p.p.b.-range mass accuracies and proteome-wide protein quantification. *Nat. Biotechnol* 2008, 26:1367-137210.1038/nbt.1511.

15. Steinmetz LM, Scharfe C, Deutschbauer AM, Mokranjac D, Herman ZS, Jones T, Chu AM, Giaever G, Prokisch H, Oefner PJ, Davis RW: Systematic screen for human disease genes in yeast. *Nat Genet* 2002, 31:400-4.

16. Thompson JD, Higgins DG, Gibson TJ: CLUSTAL W: improving the sensitivity of progressive multiple sequence alignment through sequence weighting, position-specific gap penalties and weight matrix choice. *Nucleic Acids Res* 1994, 22:4673-80PMC308517.

17. Denoeud F, Roussel M, Noel B, Wawrzyniak I, Da Silva C, Diogon M, Viscogliosi E, Brochier-Armanet C, Couloux A, Poulain J, Segurens B, Anthouard V, Texier C, Blot N, Poirier P, Ng GC, Tan KSW, Artiguenave F, Jaillon O, Aury J-M, Delbac F, Wincker P, Vivarès CP, El Alaoui H: Genome sequence of the stramenopile Blastocystis, a human anaerobic parasite. *Genome Biol.* 2011, 12:R2910.1186/gb-2011-12-3-r29.

18. Wheeler DL, Barrett T, Benson DA, Bryant SH, Canese K, Chetvernin V, Church DM, Dicuccio M, Edgar R, Federhen S, Feolo M, Geer LY, Helmberg W, Kapustin Y, Khovayko O, Landsman D, Lipman DJ, Madden TL, Maglott DR, Miller V, Ostell J, Pruitt KD, Schuler GD, Shumway M, Sequeira E, Sherry ST, Sirotkin K, Souvorov A, Starchenko G, Tatusov RL, et al.: Database resources of the National Center for Biotechnology Information. *Nucleic Acids Res* 2008, 36:D13-21PMC2238880.

19. Szklarczyk D, Franceschini A, Kuhn M, Simonovic M, Roth A, Minguez P, Doerks T, Stark M, Muller J, Bork P, Jensen LJ, von Mering C: The STRING database in 2011: functional interaction networks of proteins, globally integrated and scored. *Nucleic Acids Res* 2011, 39:D561-56810.1093/nar/gkq973.

20. Huigsloot M, Nijtmans LG, Szklarczyk R, Baars MJH, van den Brand MAM, Hendriksfranssen MGM, van den Heuvel LP, Smeitink JAM, Huynen MA, Rodenburg RJT: A mutation in c2orf64 causes impaired cytochrome C oxidase assembly and mitochondrial cardiomyopathy. *Am. J. Hum. Genet* 2011, 88:488-49310.1016/j.ajhg.2011.03.002.

21. Vogel RO, Janssen RJRJ, Ugalde C, Grovenstein M, Huijbens RJ, Visch H-J, van den Heuvel LP, Willems PH, Zeviani M, Smeitink JAM, Nijtmans LGJ: Human mitochondrial complex I assembly is mediated by NDUFAF1. *FEBS J* 2005, 272:5317-532610.1111/j.1742-4658.2005.04928.x.

22. Emanuelsson O, Brunak S, von Heijne G, Nielsen H: Locating proteins in the cell using TargetP, SignalP and related tools. *Nat Protoc* 2007, 2:953-71.

23. Waterhouse AM, Procter JB, Martin DMA, Clamp M, Barton GJ: Jalview Version 2--a multiple sequence alignment editor and analysis workbench. *Bioinformatics* 2009, 25:1189-119110.1093/bioinformatics/btp033.

24. Baughman JM, Nilsson R, Gohil VM, Arlow DH, Gauhar Z, Mootha VK: A computational screen for regulators of oxidative phosphorylation implicates SLIRP in mitochondrial RNA homeostasis. *PLoS Genet* 2009, 5:e100059010.1371/journal.pgen.1000590.

25. Guernsey DL, Jiang H, Campagna DR, Evans SC, Ferguson M, Kellogg MD, Lachance M, Matsuoka M, Nightingale M, Rideout A, Saint-Amant L, Schmidt PJ, Orr A, Bottomley SS, Fleming MD, Ludman M, Dyack S, Fernandez CV, Samuels ME: Mutations in mitochondrial carrier family gene SLC25A38 cause nonsyndromic autosomal recessive congenital sideroblastic anemia. *Nat. Genet.* 2009, 41:651-65310.1038/ng.359.

26. Mootha VK, Bunkenborg J, Olsen JV, Hjerrild M, Wisniewski JR, Stahl E, Bolouri MS, Ray HN, Sihag S, Kamal M, Patterson N, Lander ES, Mann M: Integrated analysis of protein composition, tissue diversity, and gene regulation in mouse mitochondria. *Cell* 2003, 115:629-4014651853.

27. Ishihama Y, Oda Y, Tabata T, Sato T, Nagasu T, Rappsilber J, Mann M: Exponentially modified protein abundance index (emPAI) for estimation of absolute protein amount in proteomics by the number of sequenced peptides per protein. *Mol. Cell Proteomics* 2005, 4:1265-127210.1074/mcp.M500061-MCP200.

28. Abramoff MD, Magelhaes PJ, Ram SJ: Image Processing with ImageJ. *Biophotonics International* 2004, 11:36-42.
